# SH2 domain protein E and ABL signaling regulate blood vessel size

**DOI:** 10.1371/journal.pgen.1010851

**Published:** 2024-01-08

**Authors:** Jennifer A. Schumacher, Zoë A. Wright, Diandra Rufin Florat, Surendra K. Anand, Manish Dasyani, Surya Prakash Rao Batta, Valentina Laverde, Kaitlin Ferrari, Laurita Klimkaite, Nina O. Bredemeier, Suman Gurung, Gretchen M. Koller, Kalia N. Aguera, Griffin P. Chadwick, Riley D. Johnson, George E. Davis, Saulius Sumanas

**Affiliations:** 1 Cincinnati Children’s Hospital Medical Center, Division of Developmental Biology, Cincinnati, Ohio, United States of America; 2 University of Cincinnati College of Medicine, Department of Pediatrics, Cincinnati, Ohio, United States of America; 3 Department of Biological Sciences, Miami University, Hamilton, Ohio, United States of America; 4 University of South Florida, Department of Pathology and Cell Biology, USF Health Heart Institute, Tampa, Florida, United States of America; 5 University of South Florida, Department of Molecular Pharmacology and Physiology, Tampa, Florida, United States of America; Iowa State University, UNITED STATES

## Abstract

Blood vessels in different vascular beds vary in size, which is essential for their function and fluid flow along the vascular network. Molecular mechanisms involved in the formation of a vascular lumen of appropriate size, or tubulogenesis, are still only partially understood. *Src homology 2 domain containing E (She)* protein was previously identified in a screen for proteins that interact with Abelson (Abl)-kinase. However, its biological role has remained unknown. Here we demonstrate that She and Abl signaling regulate vessel size in zebrafish embryos and human endothelial cell culture. Zebrafish *she* mutants displayed increased endothelial cell number and enlarged lumen size of the dorsal aorta (DA) and defects in blood flow, eventually leading to the DA collapse. Vascular endothelial specific overexpression of *she* resulted in a reduced diameter of the DA, which correlated with the reduced arterial cell number and lower endothelial cell proliferation. Chemical inhibition of Abl signaling in zebrafish embryos caused a similar reduction in the DA diameter and alleviated the *she* mutant phenotype, suggesting that She acts as a negative regulator of Abl signaling. Enlargement of the DA size in *she* mutants correlated with an increased endothelial expression of *claudin 5a (cldn5a*), which encodes a protein enriched in tight junctions. Inhibition of *cldn5a* expression partially rescued the enlarged DA in *she* mutants, suggesting that She regulates DA size, in part, by promoting *cldn5a* expression. SHE knockdown in human endothelial umbilical vein cells resulted in a similar increase in the diameter of vascular tubes, and also increased phosphorylation of a known ABL downstream effector CRKL. These results argue that SHE functions as an evolutionarily conserved inhibitor of ABL signaling and regulates vessel and lumen size during vascular tubulogenesis.

## Author summary

Blood vessels in different vascular beds vary in their size. Formation of the proper vessel size is critical for vascular function. However, mechanisms regulating blood vessel size are not well understood. Here we identified a gene of unknown function, called SH2 domain protein E (abbreviated as She), as one of regulators in blood vessel size. Zebrafish embryos, deficient in She function, form enlarged dorsal aorta. In contrast, embryos with increased She amounts form dorsal aorta of a reduced size. We show that She affects vessel size by regulating cell proliferation. Our results suggest that She functions by modulating signaling activity of Abelson kinase (ABL), which is commonly hyperactivated in human tumors. Human vascular endothelial cells, deficient in SHE function, formed enlarged tubes, suggesting that She function is conserved between different organisms. These results will help to identify the mechanisms that control blood vessel and lumen size and may promote therapeutic developments for vascular malformations, which often show greatly enlarged vessels.

## Introduction

Formation of a properly sized vascular lumen, or tubulogenesis, is one of the critical steps during vascular development. Blood vessels vary in size in different vascular beds, which is essential to allow the flow of fluids and blood cells along the vascular network. Various genetic mechanisms have been implicated in establishing and maintaining vascular lumen of a proper size. A family of Rho GTPases which include Rac1, Cdc42 and RhoA have well established roles in regulating cell cytoskeleton rearrangements that take place during vascular tubulogenesis [1–8]. Activity of these Rho GTPases is known to be regulated by integrin-fibronectin signaling [9]. Several other pathways, including VEGF and Notch signaling are also thought to contribute to regulation of vascular lumen size directly [10]. However, our understanding of molecular mechanisms that regulate vascular tubulogenesis is still incomplete.

Abelson (Abl) kinase signaling has diverse roles during morphogenesis such as regulating cytoskeletal organization that is important for cellular protrusions, cell migration, morphogenesis, adhesion, endocytosis and phagocytosis [11]. Chromosomal translocation of human ABL1 to the breakpoint cluster region (BCR) gene results in production of the BCR-ABL1 fusion protein in patients with different types of leukemias [12]. While numerous studies have focused on the role of BCR-ABL1 in leukemias, the role of ABL signaling during developmental morphogenesis is less understood. Intriguingly, Abl signaling is known to regulate cell-cell adhesion through Rho and Rac GTPases. Chemical inhibition of Abl signaling, or mutation of the two ABL homologs Abl1 and Arg in epithelial cell culture, results in activation of RhoA and increased actomyosin contractility, disrupting adherens junctions [13–16] In addition, Abl signaling is known to activate Rac1 in cultured human embryonic kidney cells [17]. Abl signaling is also known to promote cell proliferation in diverse cell types, such as smooth muscle cells and fibroblasts [18,19]. Previously established roles of Abl signaling in vascular endothelium include regulating angiogenesis, endothelial cell (EC) survival and vascular permeability [20–22]. Recent data also indicate that ABL signaling is increased in a cell culture model of venous malformations (VMs) and that inhibition of ABL signaling reduces lumen diameter in a VM model in vivo [23]. However, the role of ABL signaling in the formation of vascular lumen during embryogenesis has not been previously investigated.

ABL signaling is known to be regulated by various effector proteins. In a yeast two hybrid screen, two novel Abl-interacting proteins Shd and She were identified [24]. Together with a related Shb protein they comprise a separate family of SH2 proteins. Shd is known to be phosphorylated by Abl kinase in vitro, while She phosphorylation has not been previously investigated [24]. However, biological roles of these proteins remain largely unknown.

We have previously identified *she* as a novel vascular endothelial specific gene, which functions downstream of the ETS transcription factor Etv2 / Etsrp in zebrafish embryos [25]. *she* expression is enriched in arterial vessels, such as the dorsal aorta (DA). Its protein sequence is highly conserved between different vertebrates. However, its biological function has not been previously investigated in any model system.

Here we used genome editing approaches to generate zebrafish *she* genetic mutants. We show that *she* mutants display enlarged lumen of the dorsal aorta, followed by a failure of circulation and embryonic lethality. In contrast, She overexpression caused a reduction in the DA size. Chemical inhibition of Abl signaling caused a similar reduction of the DA size and reversed the DA enlargement observed in *she* mutants. Our data further suggest that She functions in part by regulating expression of *claudin 5a* in the DA. Knockdown of She in human umbilical vein endothelial cells (HUVECs) resulted in a similar enlargement of vascular tubes arguing that She function is evolutionarily conserved. In summary, our results identify novel roles for Abl signaling and She in regulating vascular tubulogenesis during embryonic development.

## Results

**Fig 1:**
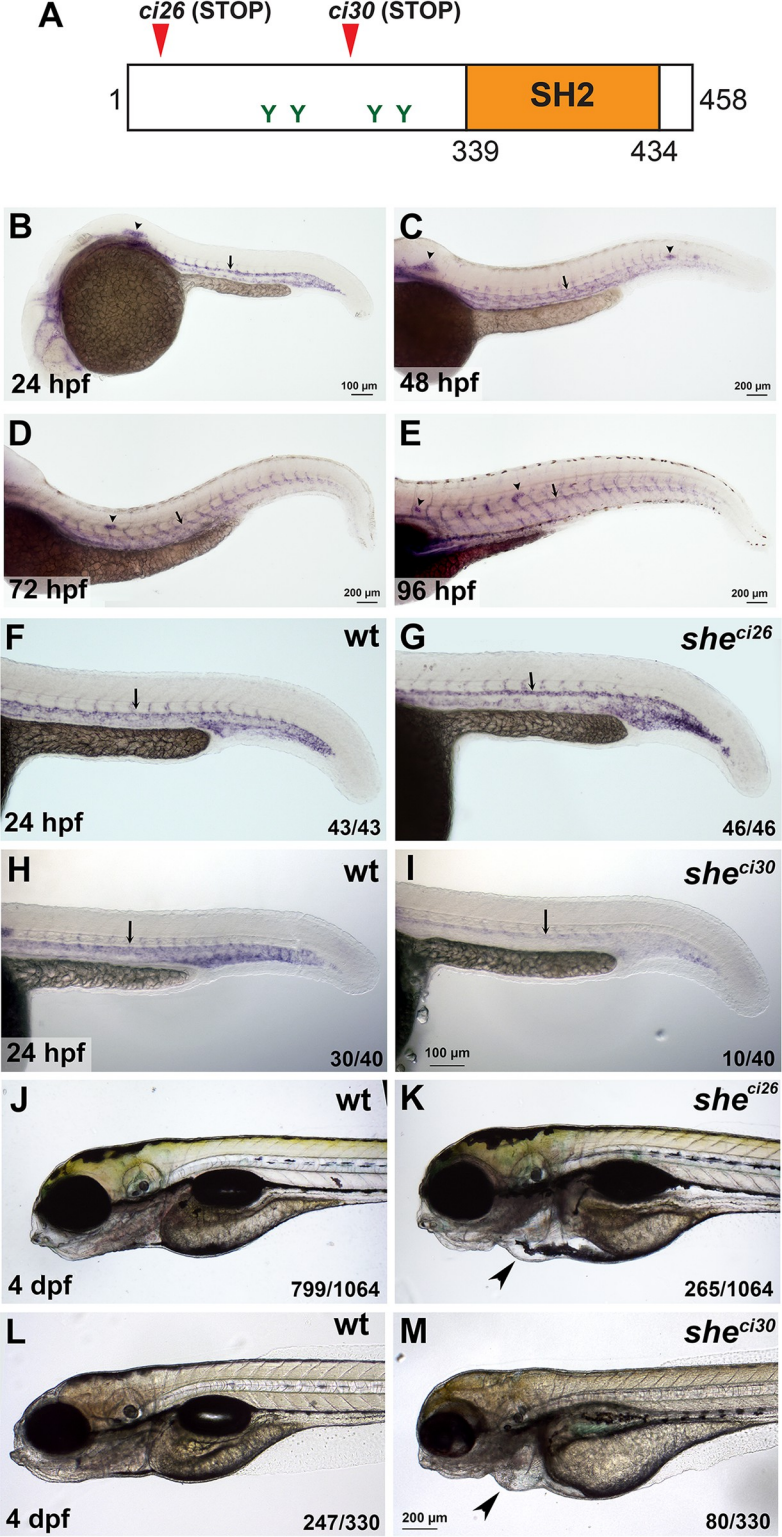
*she* mutants display pericardial edema and loss of blood circulation. (A) Zebrafish SHE protein diagram. *she ci26* and *ci30* mutant alleles are predicted to result in a frameshift and premature stop codons. SH2 domain and consensus tyrosine ABL phosphorylation sites are shown. (B-E) In situ hybridization analysis of *she* expression in wild-type embryos at 24, 48, 72 and 96 hpf stages. Note its expression in the dorsal aorta (arrows), intersegmental vessels, lateral line primordium (arrowhead, B) and neuromasts (arrowheads, C-E). (F-I) In situ hybridization analysis of *she* expression in *she* mutants at 24 hpf. Note that *she* expression is unaffected in *she*^*ci26*^ embryos while it is strongly reduced in *she*^*ci30*^ embryos. Homozygous *she*^*ci26*^ mutant embryos were obtained by an incross of *she*^*ci26-/-*^*; fli1a*:*she-2A-mCherry; kdrl*:*GFP* parents (which are viable due to *fli1a*:*she-2A-mCherry* rescue) and selected for mCherry negative embryos. Wild-type control embryos were obtained by incross of sibling wt; *fli1a*:*she-2A-mCherry* parents and selected for mCherry-negative embryos. *she*^*ci30*^ embryos were obtained by incross of *she*^*ci30+/-*^ parents and genotyped after in situ hybridization. 25% (10 out of 40) embryos showed strong reduction in *she* expression which correlated with the mutant phenotype. (J-M) Brightfield images of *she*^*ci26*^ and *she*^*ci30*^ mutant embryos and their siblings (wild-type and or heterozygous) at 4 dpf. Note the pericardial edema (arrowheads) in the mutant embryos. Embryos were obtained by the incross of heterozygous parents in *kdrl*:*GFP* background. 24.9% (265 out of 1064) and 24.2% (80 out of 330) embryos obtained in *she*^*ci26*^ or *she*^*ci30*^ incross, respectively, showed this phenotype. A subset of embryos was genotyped to confirm the correlation between the phenotype and genotype.

Our previous data showed that *she* is expressed in vascular endothelium in zebrafish embryos and is enriched in arterial vessels, in particular the dorsal aorta at 24 hours-post-fertilization (hpf) and younger stages [25]. To test if a similar expression pattern continues at later stages, we performed in situ hybridization analysis (ISH) at 1–4 days-post-fertilization (dpf). *she* expression at these stages was also enriched in arterial vessels, including the dorsal aorta (Fig 1B–1E). In addition, *she* expression was apparent in the lateral line primordium and neuromasts. To determine its function in cardiovascular development, we used TALEN and CRISPR/Cas9 genome editing to generate two loss-of-function *she* mutant alleles (S1 Fig). The *she*^*ci26*^ allele has a 7 bp deletion, which is predicted to result in a frameshift after amino acid 4 and is expected to lead to premature translation termination. The *she*^*ci30*^ allele has a deletion of 575 bp and an insertion of 24 bp which is predicted to lead to a frameshift and a premature stop codon after amino acid 243 (Figs 1A and S1). *she* mRNA expression in *she*^*ci26*^ mutants was not significantly affected, while *she* mRNA expression in *she*^*ci30*^ mutant embryos was significantly decreased (Fig 1F–1I), indicating a possibility of nonsense-mediated RNA decay.

Homozygous *she* mutant embryos were then obtained by an incross of respective heterozygous adult carriers and analyzed for morphological defects. The embryos appeared morphologically normal until 4 days-post-fertilization (dpf). At 4–6 dpf both *she*^*ci26*^ and *she*^*ci30*^ mutants developed pericardial edema, and their blood circulation slowed down or completely stopped (Fig 1J–1M and S1 and S2 Movies). As a result, mutant embryos for both alleles were lethal by approximately 7 dpf. *she*^*ci26*^ mutants showed edema and blood circulation failure at a slightly earlier stage (4 dpf) compared to the *she*^*ci30*^ allele (5–6 dpf). *she*^*ci26*^ and *she*^*ci30*^ mutants failed to complement each other, and in the complementation cross 58 out of 262 embryos (22.1%) developed pericardial edema and greatly reduced or absent blood flow by 6 dpf. Because *she*^*ci26*^ allele showed a slightly more severe phenotype, it was used for all further studies.

**Fig 2:**
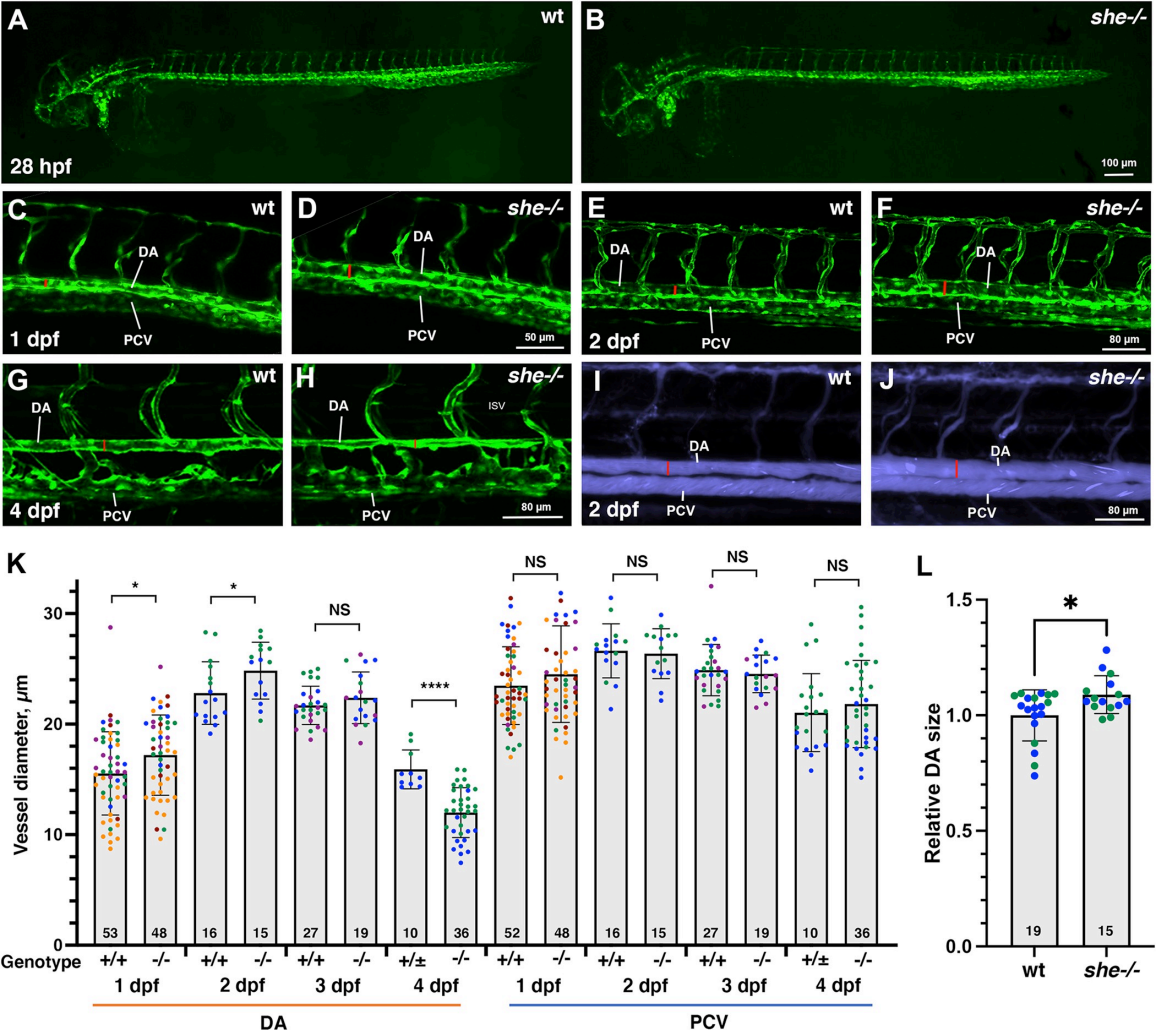
*she* mutants show enlarged diameter of the dorsal aorta. (A,B) Overall vascular patterning of *she-/-* mutants is normal when compared to their sibling wild-type embryos at 28 hpf. Embryos are in *kdrl*:*GFP* background. (C-F) A wider DA is observed in *she* mutant embryos compared to their wild-type *(she+/+)* siblings at 1 and 2 dpf (28 and 48 hpf respectively). Red line indicates DA diameter. (G,H) DA is narrower in *she* mutants at 4 dpf compared to their siblings. (I,J) Qtracker dots were injected into the circulatory system at 2 dpf (48 hpf) stage. Wider DA is apparent in *she* mutants (red lines), indicating enlarged vascular lumen size. (K) Diameter of the DA and PCV at 1–4 dpf in *she* mutants and their wild-type siblings. * p<0.05; ****p<0.0001, NS–not significant, Student’s t-test. Error bars show SD. Data were combined from 2 (2 dpf and 4 dpf), 3 (3 dpf) or 5 (1 dpf) replicate experiments; data points from each replicate experiment are shown in different colors. (L) Quantification of DA lumen size based on the imaging of embryos injected with Qtracker dots. Relative DA size was calculated by dividing each value over an average lumen size in wt embryos. *p<0.05, Student’s t-test. Data were combined from 2 replicate experiments, shown in different colors. Error bars show SD. At all stages, s*he* mutant and sibling embryos were obtained by in-crossing *she*^*ci26+/-*^; *kdrl*:*GFP* carriers. Embryos at 1 and 2 dpf were genotyped after imaging. Embryos at 4 dpf were separated based on the phenotype, and wild-type siblings include *she+/+* and *she+/-* embryos at this stage. Numbers at the bottom of the bars indicate the total number of embryos analyzed.

To analyze potential vascular defects, *she* mutants were crossed into vascular endothelial reporter *kdrl*:*GFP* background. Overall vascular patterning was normal and unaffected at 1–4 dpf, when analyzed by confocal imaging for GFP expression (Fig 2A and 2B). In addition, arterial and venous trunk intersegmental vessel sprouting appeared normal in mutant and wild-type sibling embryos (S2 Fig). There was also no effect on endothelial *kdrl*, arterial *flt1* or venous marker *dab2* expression when analyzed by in situ hybridization at 24 hpf (S3 Fig). However, the size of the dorsal aorta (DA) was slightly enlarged in *she* mutants compared with their sibling wild-type (wt) embryos at both 1 and 2 dpf (Fig 2C–2F and 2K). Notably, no circulation defects were apparent in *she* mutants at these stages. In contrast, the DA was narrower in *she* mutants at 4 dpf when compared to their siblings (Fig 2G, 2H and 2K). It is likely that the DA collapse apparent at this stage is a secondary consequence of failing blood circulation. The diameter of the posterior cardinal vein (PCV) was not significantly different between *she* mutant and wild-type embryos at 1–4 dpf (Fig 2K). To confirm if changes in the DA size observed by *kdrl*:*GFP* fluorescence correlate to vascular lumen size, we injected fluorescently labeled quantum dots (Q-dots) into the circulatory system of wt and *she* mutant embryos at 48 hpf stage. *she* mutants exhibited larger DA lumen compared to wt siblings (Fig 2I, 2J and 2L). This suggests that She may function to restrict the DA lumen size during vascular development.

Blood flow is known to induce expression of many flow-dependent genes [26]. To determine if *she* expression and *she* mutant phenotype is dependent on blood flow, we injected the previously validated morpholino (MO) against Cardiac muscle troponin T (Tnnt2), which mediates heart muscle contraction [27]. *tnnt2* MO-injected embryos showed no circulation, yet *she* expression was unaffected (S4A and S4B Fig). Due to the absence of circulation, DA was collapsed and much narrower in *tnnt2* MO-injected embryos. Intriguingly, even in the absence of circulation, the DA was larger in *she* mutant embryos, injected with *tnnt2 MO*, compared to wild-type *tnnt2* MO-injected sibling embryos (S4C–S4H Fig). These results argue that the DA enlargement, observed in *she* mutants, does not depend on blood flow.

**Fig 3:**
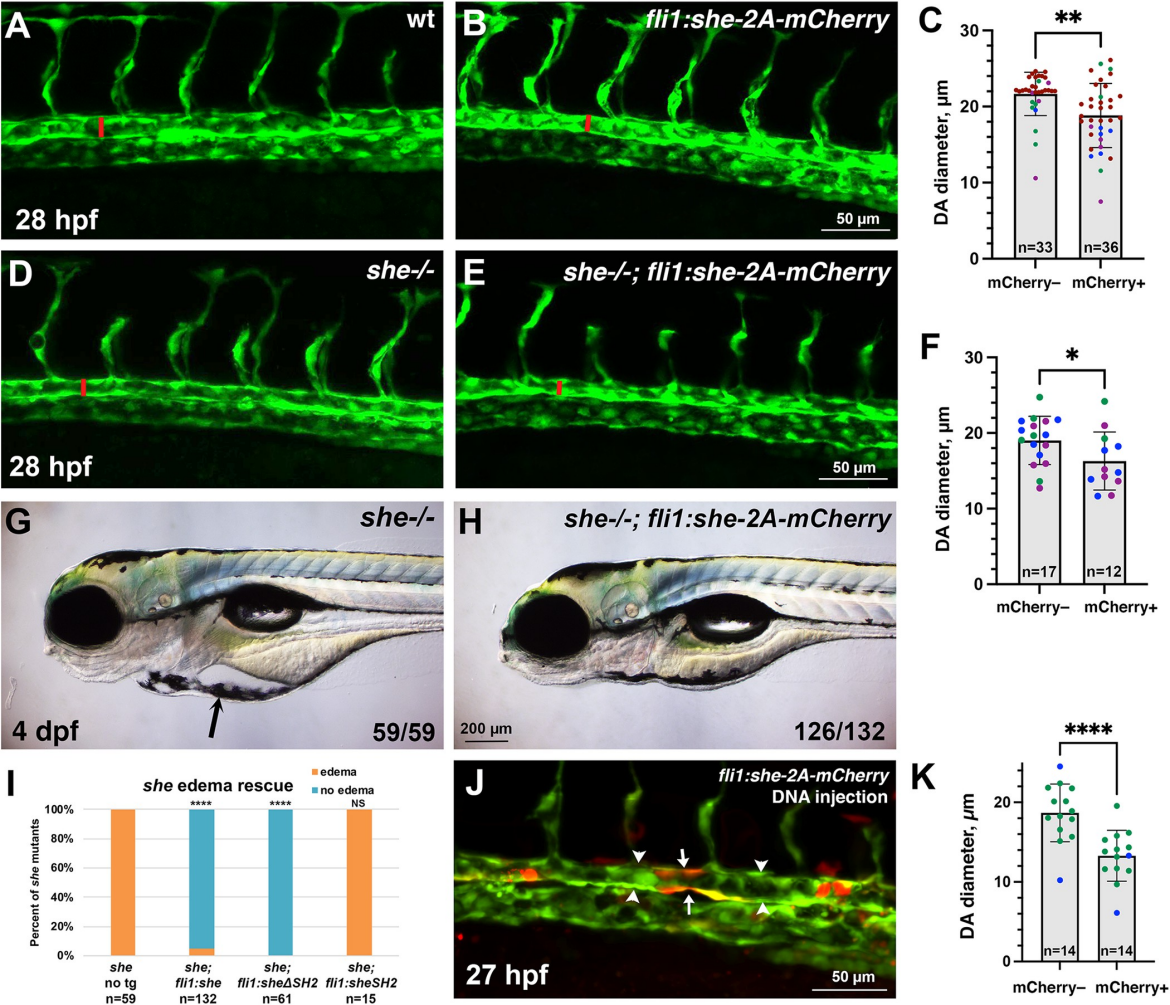
*she* overexpression in vasculature reduces the diameter of the dorsal aorta and rescues the *she* mutant phenotype. (A-C) Quantification of trunk vasculature in wild-type and *fli1*:*she-2A-mCherry* embryos in *kdrl*:*GFP* background at 28 hpf. Confocal imaging of GFP expression in live embryos obtained from stable transgenic lines; the diameter of the DA is marked with red line. Note the reduction of DA diameter in *fli1*:*she-2A-mCherry* embryos compared to wild-type non-transgenic (mCherry-) siblings. Embryos were obtained by incross of *she+/+* (referred to as wild-type); *fli1*:*she-2A-mCherry+/-* parents and separated based on mCherry expression. (D-F) Quantification of trunk vasculature in *she-/-* and *she-/-; fli1*:*she-2A-mCherry* embryos at 28 hpf. The diameter of the DA is marked with red line. Note that the DA diameter is reduced in *she-/-; fli1*:*she-2A-mCherry* embryos compared to *she* siblings without the transgene (mCherry-). Embryos were obtained by incross of *she-/-; fli1*:*she-2A-mCherry+/-* parents and separated based on mCherry expression. Note that wild-type and *she-/-* mCherry- embryos in (C and F) cannot be directly compared in this experiment because they came from different parents and are not siblings; embryos from different pairs can show significant variability in the DA diameter. (G,H) Pericardial edema in *she-/-* mutants (G) is rescued in *she-/-; fli1*:*she-2A-mCherry* embryos at 4 dpf. (I) Percentage of *she* mutant embryos showing pericardial edema at 4 dpf. Note that vascular endothelial expression of full length She (*fli1*:*she-2A-mCherry)* and the construct carrying a deletion of the SH2 domain in She protein *(fli1*:*sheΔSH2-2A-mCherry)* rescues the mutant phenotype, while overexpression of She construct with SH2 domain alone *(fli1*:*sheSH2-2A-mCherry)* fails to rescue the phenotype. ****p<0.0001, NS–not significant, Fisher’s exact test, compared to *she* no transgene embryos. (J) Injection of *fli1*:*she-2A-mCherry* DNA plasmid together with *tol2* mRNA results in mosaic expression of *she-2A-mCherry* in endothelial cells. Note that mCherry positive segments of the DA (arrows) show reduced diameter compared to adjacent mCherry-negative segments (arrowheads). (K) Quantification of the DA diameter in mCherry+ and mCherry–DA segments. In all graphs, error bars show SD; *p<0.05, **p<0.01, ****p<0.0001, Student’s t-test. 3 replicate experiments were performed in A-H, and 2 replicate experiments were performed in I-K; data points from separate replicate experiments are shown in different colors.

To test if She function is sufficient to limit the size of the DA, we made a construct which contains the coding sequence for the zebrafish She driven by the vascular endothelial *fli1a* promoter, followed by the self-cleaving peptide 2A and mCherry reporter. A stable zebrafish transgenic reporter line was then established using Tol2-mediated transgenesis (S5A–S5C Fig). *fli1*:*she-2A-mCherry* embryos were viable, yet they exhibited a reduction in the DA diameter at 28 hpf, when compared to sibling embryos without the transgene (Fig 3A–3C). To test if vascular endothelial *she* expression could rescue the *she* mutant phenotype, *fli1*:*she-2A-mCherry* line was crossed into the *she*^*ci26*^ mutant background. Remarkably, *she-/-; fli1*:*she-2A-mCherry* embryos were viable and had smaller DA diameter when compared with *she-/-* sibling embryos without the transgene (Fig 3D–3F). They did not exhibit pericardial edema and showed normal blood circulation at 4 dpf (Fig 3G–3I). These results argue that She functions in the vascular endothelium to regulate the DA size.

SH2 domains are known to bind to phosphorylated tyrosines [28]. To test the functional requirement for the SH2 domain within the zebrafish She protein, we created She deletion constructs (S5D Fig) and generated stable zebrafish transgenic lines using vascular endothelial *fli1* promoter. Intriguingly, the construct that lacked the SH2 domain was sufficient to rescue the *she* mutant phenotype, and no pericardial edema or circulation defects were observed in *fli1*:*sheΔSH2-2A-mCherry; she-/-* embryos, which were viable through adulthood (Fig 3I). In contrast, the construct containing only the SH2 domain was insufficient to rescue the *she* mutant phenotype (Fig 3I).

To test if She function is required cell-autonomously, we injected full-length *fli1*:*she-2A-mCherry* DNA construct in combination with *tol2* mRNA into zebrafish embryos at 1-cell stage, which resulted in mosaic overexpression of *she-2A-mCherry* in vascular endothelial cells. Interestingly, segments of the DA with multiple mCherry-positive cells displayed narrower size compared with adjacent mCherry-negative regions of the DA (Fig 3J and 3K). This argues that She function is required cell-autonomously within vascular endothelial cells.

While DA enlargement was observed at 1 dpf, blood circulation defects and pericardial edema were not apparent until 4 dpf in *she* mutants. To determine when She function is required to maintain normal circulation, we made a construct which encodes full-length zebrafish She under the heat-shock inducible *hsp70* promoter, followed by 2A-mCherry and established stable zebrafish lines using Tol2 transgenesis. Heat-shock at 3 or 3.5 dpf was sufficient to rescue the pericardial edema phenotype fully or partially in *she* mutant embryos, positive for the *hsp70*:*she-2A-mCherry* transgene (S6A and S6B Fig). This also prevented the collapse of DA at 4 dpf (S6C–S6H Fig). This suggests that She has a critical functional requirement between 3 and 4 dpf.

**Fig 4:**
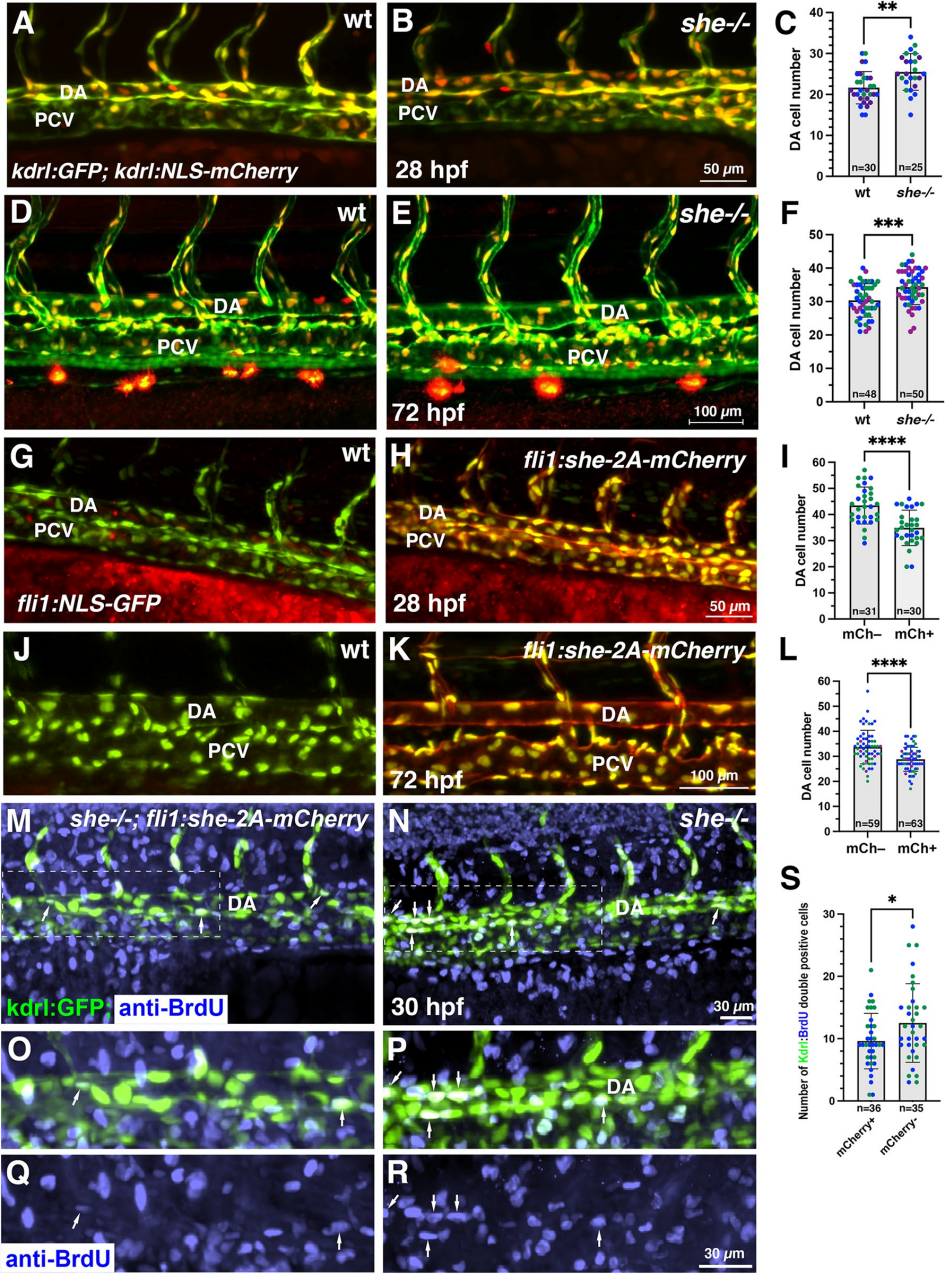
*she* affects cell number and inhibits endothelial cell proliferation. (A-F) Analysis of cell number in the DA of *she* mutant and wild-type *(she+/+)* sibling embryos at 28 and 72 hpf. Embryos were obtained from an incross of *she+/-; fli1*:*GFP; kdrl*:*NLS-mCherry* parents, imaged by confocal microscopy and subsequently genotyped. Note increased cell number in *she* mutant embryos. (G-L) Analysis of cell number in the DA of *fli1*:*she-2A-mCherry; fli1*:*NLS-GFP* embryos and their mCherry negative (wt) siblings at 28 and 72 hpf. Note the reduced cell number in mCherry+ embryos. (M-S) Cell proliferation analysis using BrdU incorporation assay in *she-/-; fli1*:*she-2A-mCherry* embryos (phenotypically normal) and their sibling mCherry negative *she-/-* embryos in *kdrl*:*GFP* background at 30 hpf. (O-R) show enlarged view of the area in insets (M,N). Note the increased number of BrdU and *kdrl*:*GFP* double positive cells within the DA in mCherry-negative *she* mutant embryos. All graphs show data combined from 2 (I,S) or 3 (C,F,L) independent experiments; data points from different experiments are shown in distinct colors. Mean±SD is shown. *p<0.05, **p<0.01, ***p<0.001, ****p<0.0001, Student’s t-test.

To determine the cellular cause of the enlarged aorta observed in *she* mutants, we analyzed the endothelial cell number. The number of cells in the DA was significantly increased in *she* mutants crossed to the *kdrl*:*NLS-mCherry* endothelial nuclear reporter line when compared with their wild-type siblings at 28 and 72 hpf stages (Fig 4A–4F). In contrast, *fli1*:*she-2A-mCherry* embryos, which overexpress *she* in endothelial cells, had significantly fewer cells in the DA compared to their sibling mCherry negative embryos in the nuclear *fli1*:*NLS-GFP* reporter background at 28 and 72 hpf (Fig 4G–4L). As analyzed by BrdU incorporation assay at 30 hpf, *fli1*:*she-2A-mCherry* embryos in *she* mutant background (which are phenotypically normal) showed reduced cell proliferation within the DA compared with mCherry-negative *she*-/- sibling embryos (Fig 4M–4S). These results argue that *she* restricts the DA size by inhibiting endothelial cell proliferation. We also analyzed mural cell formation by performing fluorescent ISH analysis for *pdgfrb* expression. However, no significant change in *pdgfrb* expression was observed, suggesting that the changes in the DA size are not caused by defects in mural cell formation (S7 Fig).

**Fig 5:**
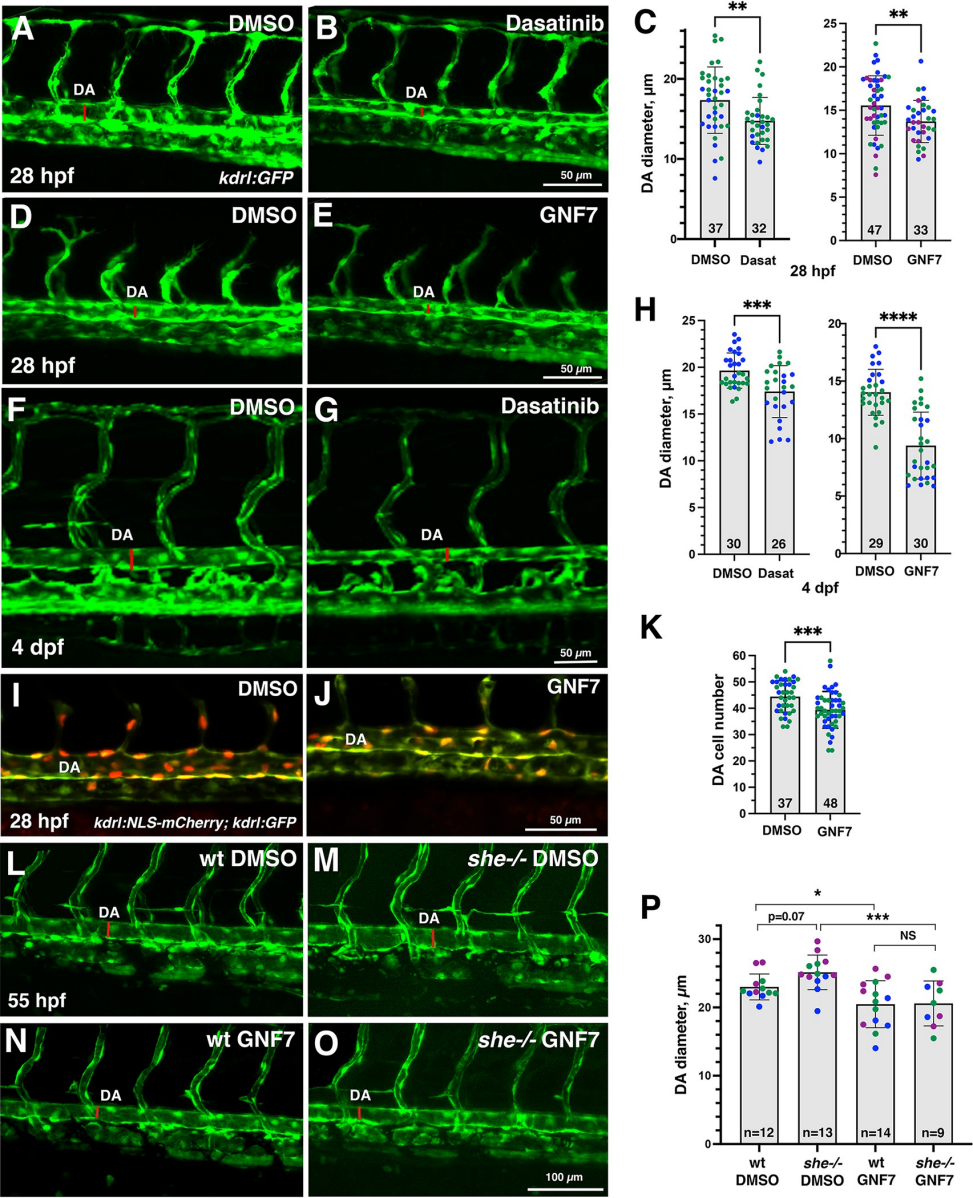
Inhibitors of Abl signaling reduce DA diameter in wild-type and *she* mutant embryos. (A-E) Dorsal aorta diameter at 28 hpf is reduced in *kdrl*:*GFP* embryos treated with 5 μM Dasatinib or 1 μM GNF-7 compared to controls treated with 0.1% DMSO. (F-H) Embryos treated with 5 μM Dasatinib (F-H, left) or 1 μM GNF-7 (H, right) exhibit narrower DA at 4 dpf compared to controls treated with 1% DMSO (GNF-7) or 2% DMSO (Dasatinib treatments). (I-K) The number of cells in the DA is reduced in embryos at 28 hpf treated with 1 μM GNF-7 compared to control embryos treated with 0.1% DMSO. (L-O) GNF-7 treatment reverses DA enlargement in *she* mutant embryos. *she+/-; kdrl*:*GFP* adults were crossed to obtain *she* mutant embryos. Embryos were treated starting at 6 hpf with either 0.5 μM GNF-7 or 0.1% DMSO. Embryos were imaged at approximately 55 hpf and subsequently genotyped. DA measurements were performed blinded. Mid-trunk region is shown, anterior is to the left. Note the slightly wider DA (red line) in *she-/-* mutant embryos compared to wild-type *(she+/+)* siblings. DA is reduced in both wild-type and *she* mutant embryos treated with GNF-7. (P) Quantification of DA diameter in wild-type or *she* mutant embryos treated with GNF-7 or DMSO. In all graphs mean±SD is shown. Data points (shown in different colors) are combined from 2 (left graph C,H,K) or 3 (right graph C,P) independent experiments. Total number of embryos analyzed is shown at the bottom of each bar. *p<0.05, **p<0.01, ***p<0.001, ****p<0.0001, NS–not significant, Student’s t-test (C,H,K), or one-way ANOVA test, followed by multiple comparisons Fisher’s LSD (P).

It has been previously demonstrated that SHE interacts with ABL kinase in vitro [24]. To test a potential role for ABL signaling in regulating vascular lumen size, we treated zebrafish embryos with chemical inhibitors of ABL signaling, dasatinib and GNF-7 [29,30], starting at 6 hpf. Embryos treated with either ABL inhibitor displayed reduction in the DA size at 28 hpf (Fig 5A–5E). No other morphological defects were observed at this stage. By 4 dpf dasatinib and GNF-7-treated embryos developed pericardial edema and their blood circulation slowed down or stopped, while the DA diameter remained reduced at this stage (Fig 5F–5H). Importantly, the number of endothelial cells in the DA was reduced in GNF7 treated embryos at 28 hpf, compared to DMSO-treated controls (Fig 5I–5K).

These results show that both She overexpression and inhibition of Abl signaling resulted in similar phenotypes of reduced DA diameter and lowered arterial cell number. We tested if inhibition of Abl signaling may reverse the enlarged DA observed in *she* mutants. *she* mutants and wild-type sibling embryos were treated with GNF-7 and analyzed for the DA diameter at 2 dpf. Indeed, while *she* mutants showed enlarged DA, GNF-7 treatment reversed this phenotype and resulted in a reduced width DA (Fig 5L–5P).

Two zebrafish homologs of *ABL* gene, *abl1* and *abl2* are present in the zebrafish genome. Both homologs are expressed in many cell types during zebrafish embryogenesis, including vascular endothelial cells, based on the analysis of published single-cell RNA-seq datasets [31] (S8 Fig). No previous mutant analysis of zebrafish *abl* genes has been reported. We designed 3 single-guide RNAs against each *abl* gene, targeting different coding exons and injected into zebrafish embryos together with Cas9 protein. Embryos injected with the mixture of *abl1* and *abl2* gRNAs showed reduced DA size, similar to the chemical inhibitor treated embryos (S9 Fig). These results provide further support for the requirement of ABL signaling in regulating the DA size.

**Fig 6:**
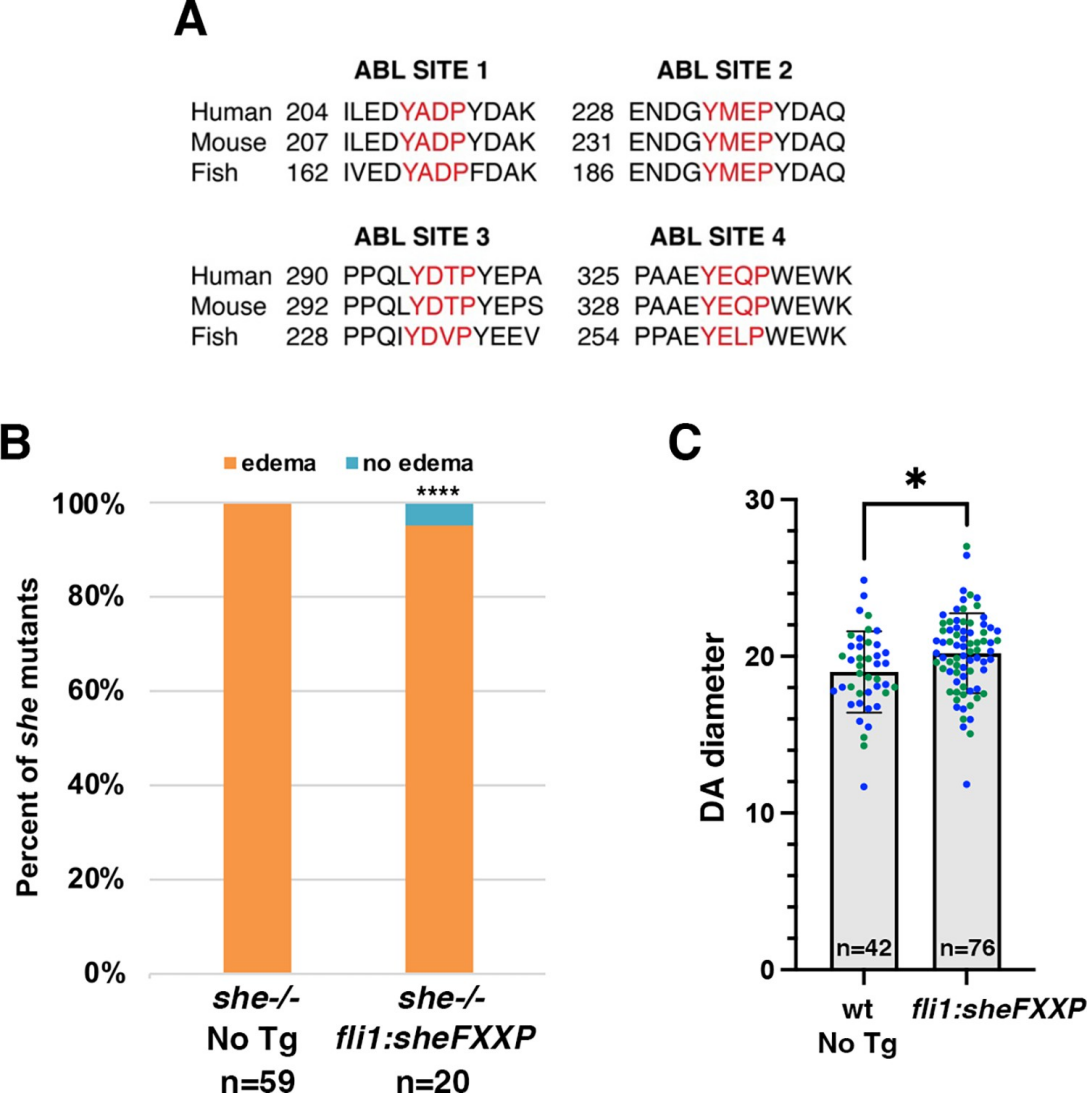
Consensus ABL phosphorylation sites YXXP are required for SHE function. (A) Consensus ABL phosphorylation sites are conserved between zebrafish, mice and humans. (B) Vascular endothelial expression of a mutant construct *fli1*:*sheFXXP-2A-mCherry*, where all four consensus tyrosines have been substituted into phenylalanine, fails to rescue the pericardial edema in *she* mutants at 4 dpf. The first bar showing *she-/-* embryos (no Tg) is copied from Fig 3I. ****p<0.0001, Fisher’s exact test. (C) DA diameter measurement (μm) in *fli1*:*sheFXXP-2A-mCherry* embryos and sibling mCherry-negative embryos at 28 hpf. 5 measurements were performed in each embryo, which were then averaged for statistical calculations. 2 replicate experiments were performed, shown in different colors. n corresponds to the number of embryos. Mean ± SD is shown. *p<0.05, Student’s t-test.

It has been previously shown that ABL kinase can phosphorylate a related protein SHD in vitro [24]. The SHE sequence has four consensus tyrosine kinase phosphorylation sites with the YXXP motif (Fig 6A), which are conserved between different vertebrates and between SHE and SHD. Therefore, we hypothesized that ABL kinase may similarly target SHE protein for phosphorylation. To test the in vivo requirement for the consensus phosphorylation sites, we performed site directed mutagenesis on the zebrafish She to substitute all four consensus tyrosines into phenylalanine (YXXP–> FXXP). A zebrafish line was established that expressed the mutated She FXXP sequence under *fli1* promoter. Expression of the mutant construct failed to rescue pericardial edema and blood circulation defects in *she* mutant embryos (Fig 6B). In contrast to overexpression of wild-type *fli1*:*she-2A-mCherry* construct which results in narrower DA, *fli1*:*sheFXXP-2A-mCherry* embryos showed DA enlargement at 28 hpf stage (Fig 6C). Overexpression of the mutant SheFXXP may interfere with the wild-type She protein, resulting in a dominant negative effect, which can explain the DA enlargement observed in *fli1*:*sheFXXP* embryos. Altogether, these results argue that the consensus YXXP phosphorylation sites are required for SHE function.

**Fig 7:**
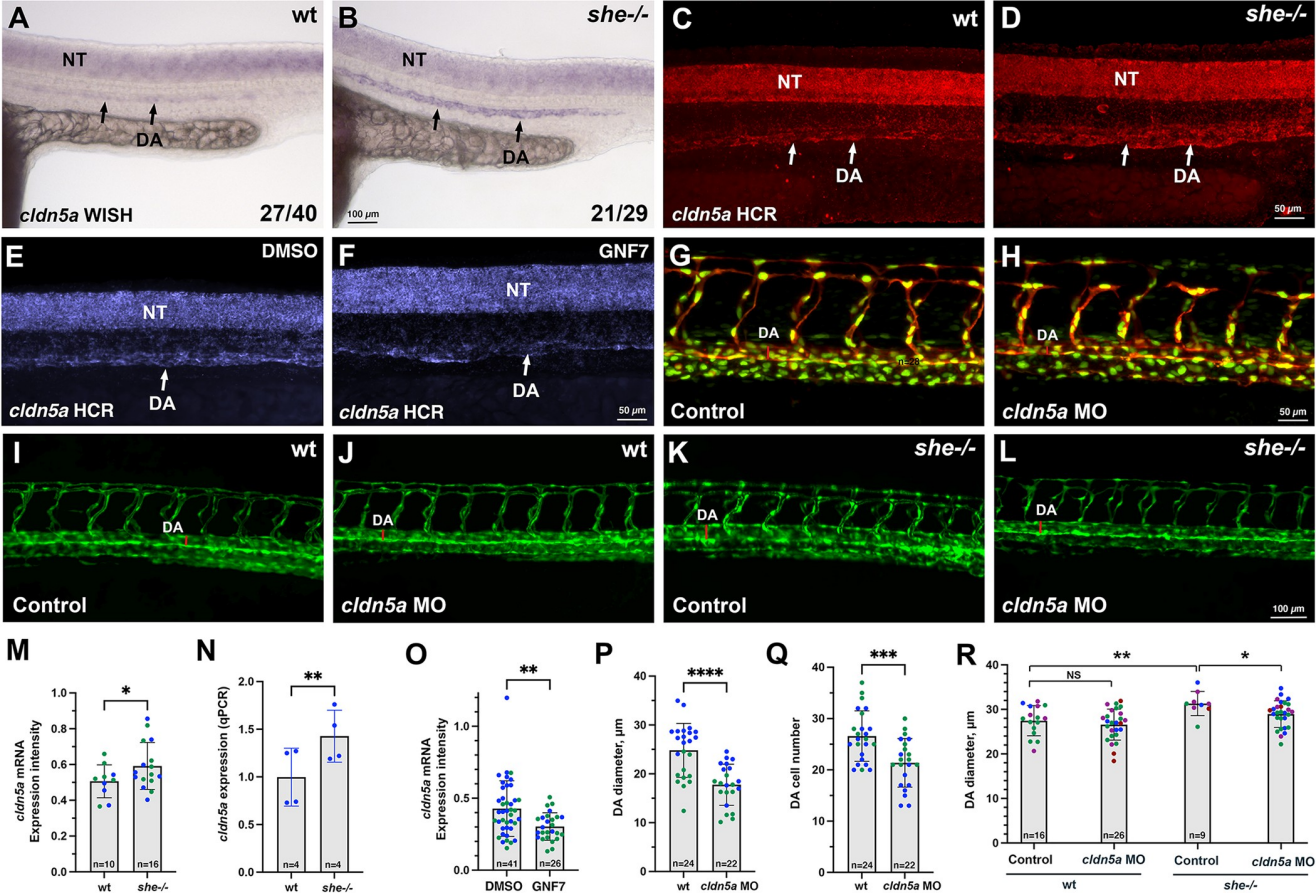
She regulates vascular lumen size by inhibiting *cldn5a* expression. (A-D) Chromogenic whole mount in situ hybridization (WISH) (A,B) and fluorescent in situ hybridization analysis using hybridization chain reaction (HCR) (C,D) analysis for *cldn5a* expression in *she* mutants and sibling wild-type *(she+/+)* embryos at 24 hpf. Note increased *cldn5a* expression in the dorsal aorta (DA) in *she* mutants while neural tube (NT) expression is unaffected. Quantification of *cldn5a* expression in the DA is shown in (M). Embryos were obtained by incross of heterozygous *she* parents and genotyped after WISH or HCR. (E,F) HCR analysis for *cldn5a* expression in control 0.01% DMSO or 1 μM GNF7 treated *kdrl*:*GFP* embryos. Trunk region is shown, anterior is to the left. Note the reduction in *cldn5a* expression in the DA while neural tube expression is unaffected. Quantification is shown in (O). (G,H) 2 ng *cldn5a* MO injection reduces DA size (red line across the DA) and DA cell number in *kdrl*:*mCherry; fli1*:*NLS-GFP* embryos at 28 hpf. Quantification is shown in (P,Q). (I-L) 1 ng dose *cldn5a* MO injection reduces enlarged DA in *she* mutants. Embryos were obtained by an incross of *she+/-; kdrl*:*GFP* parents and imaged for GFP expression at 2 dpf. Mid-trunk region is shown, anterior is to the left. Quantification is shown in (R). Note the increase in DA width (red line) in *she* mutants (K), which is reduced upon *cldn5a* MO injection (L). (M) Quantification of *cldn5a* expression in the DA of *she* mutants and wild-type siblings after HCR fluorescent in situ hybridization at 24 hpf. To reduce the staining variability between different embryos, expression values in the DA were normalized to the neural tube expression in each embryo. Expression analysis was performed blindly without knowledge of embryo genotypes. Datapoints have been combined from two independent experiments, shown in different colors. Mean±SD shown. *p<0.05, Student’s t-test. (N) qPCR analysis of *cldn5a* expression in FACS-sorted vascular endothelial cells obtained from *she* mutant and wild-type embryos at 24 hpf. Expression was normalized to the house-keeping *EF1a* gene. Embryos were obtained by incross of *she-/-; fli1*:*she-2A-mCherry+/-* or *she+/+* (wild-type); *fli1*:*she-2A-mCherry+/-* parents in *kdrl*:*GFP* background and sorted for the absence of mCherry. Mean±SD shown. **p<0.01, Student’s t-test. (O) Quantification of *cldn5a* expression in embryos treated with 0.01% DMSO or 1 μM GNF7 at 24 hpf. Relative expression in the DA was calculated by dividing the intensity in the DA over the expression intensity in the neural tube. Datapoints have been combined from two independent experiments, shown in different colors. Error bars show mean±SD. **p<0.01, Student’s t-test. (P,Q) Quantification of DA diameter (P) and DA cell number (Q) in embryos injected with 2 ng of *cldn5a* MO. Datapoints were combined from two independent experiments, shown in different colors. Mean±SD shown. ***p<0.001, ****p<0.0001, Student’s t-test. (R) Quantification of DA diameter at 2 dpf in wild-type and *she* mutant embryos injected with 1 ng *cldn5a* MO. Mean±SD shown. *p<0.05, ** p<0.01, NS–not significant, one-way ANOVA analysis, followed by multiple comparisons Fisher’s LSD test.

Recently it has been reported that zebrafish mutants for claudin *cldn5a*, the homolog of the mammalian Cldn5, exhibit reduced DA [32]. Therefore, we tested if *cldn5a* expression was altered in *she* mutants. Indeed, *she* mutants exhibited increased *cldn5a* expression within the DA at 24 hpf when analyzed by standard chromogenic and fluorescent hybridization chain reaction (HCR) in situ hybridization approaches (Fig 7A–7D). Average *cldn5a* fluorescence intensity was increased by 17% in *she* mutants compared to wild-type embryos (Fig 7M). This increase was not due to DA enlargement because the same size area within the DA was used to measure *cldn5a* expression level in *she* mutant and wild-type sibling embryos (see Materials and Methods). Expression of *cldn5a* in the neural tube was unaffected in *she* mutants. To verify this result, we purified all vascular endothelial cells using FACS sorting from *kdrl*:*GFP* line in *she* mutants and wild-type embryos at 24 hpf and performed qPCR. After normalization,1.4-fold increase in *cldn5a* expression was observed using this approach (Fig 7N). Increased expression trend for a related *cldn5b* homolog was also apparent, although it did not reach statistical significance (S10 Fig). In contrast, inhibition of Abl signaling by treating wild-type *kdrl*:*GFP* embryos with GNF7 resulted in a reduced *cldn5a* expression in the DA (Fig 7E,7F, and 7O). To test if reduction in *cldn5a* level could restore the DA size, we knocked down *cldn5a* function in *she* mutants and sibling embryos using the previously validated Cldn5a morpholino [32]. Injection of full 2 ng MO dose caused reduction in the DA size, previously reported in *cldn5a* mutants, which also correlated with the reduced cell number in the DA at 28 hpf stage (Fig 7G, 7H, 7P and 7Q). A subphenotypic dose of MO was then used, which did not result in a significant reduction of DA width in wild-type embryos. Intriguingly, the same dose of *cldn5a* MO resulted in a reduction of DA diameter in *she* mutants leading to a partial rescue of the *she* phenotype (Fig 7I–7L and 7R).

**Fig 8:**
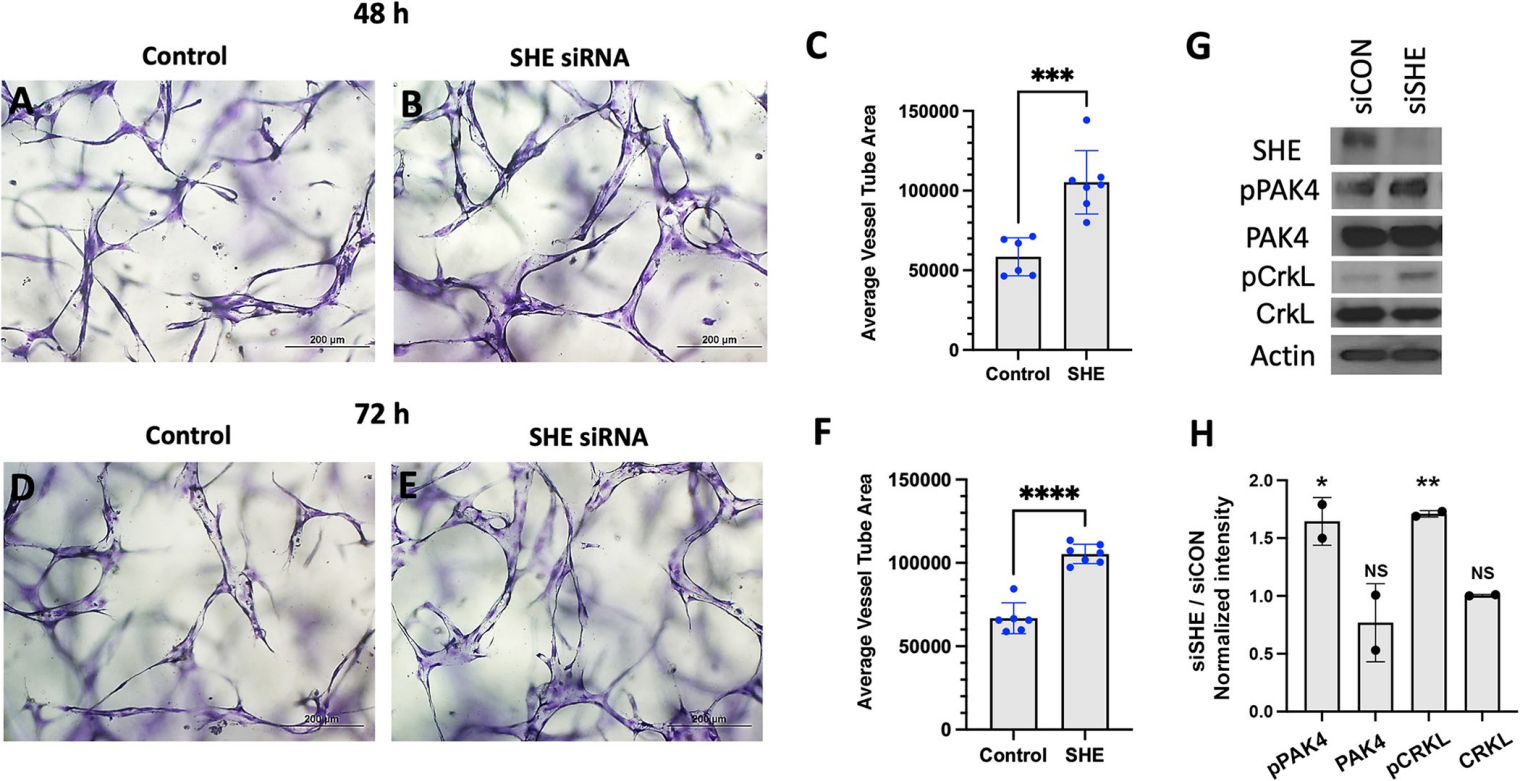
Inhibition of SHE in HUVECs results in enlarged tubulogenesis. (A-F) HUVEC cells were transfected with either control or SHE siRNA and analyzed in 3D collagen matrix assay at 48 (A-C) or 72 h (D-F) after transfection. The values (± standard deviation) are derived from 6–7 representative fields from 3 replicate wells where total EC tube area was measured. *** p<0.001; ****p<0.0001, Student’s t-test. (G) Western blots for expression of SHE and phosphorylation of PAK4, and CRKL in HUVEC cells transfected with a control or SHE siRNA. Note greatly reduced SHE expression and increased pPAK4 and pCRKL in cells transfected with siSHE RNA. Analysis was done at 48hr after transfection except for pCRKL and CRKL, which are 72 hr. Two replicate experiments were performed; full results are shown in S11 Fig. (H) Relative intensity ratio in siSHE / siControl samples. Note increased intensity in pPAK4 and pCRKL samples compared to siControl (which equals to 1); *p<0.05, **p<0.01, Student’s t-test.

To test the conservancy of She function in mammalian cells, we inhibited SHE function in human umbilical vein endothelial cells (HUVECs) using siRNA. Cells were transfected with either SHE or control siRNA, and tubulogenesis was assayed using 3D collagen matrix assay under serum-free defined conditions. siRNA transfected cells showed enlarged vascular tubes compared to control cells, reproducibly observed both at 48 and 72 hours after transfection (Fig 8A–8F). Assay of intracellular signaling kinases by Western blot showed increased phosphorylation of pPAK4 (Figs 8G, 8H, and S11), which is a known signaling effector acting downstream of Cdc42 during vascular tubulogenesis [7,8,33]. Importantly, phosphorylation of a known ABL signaling effector CRKL was also increased in SHE inhibited cells (Figs 8G, 8H, and S11). These results argue that She function is conserved between vertebrates and suggest that She restricts blood vessel size by inhibiting Abl and Cdc42 activities.

## Discussion

**Fig 9:**
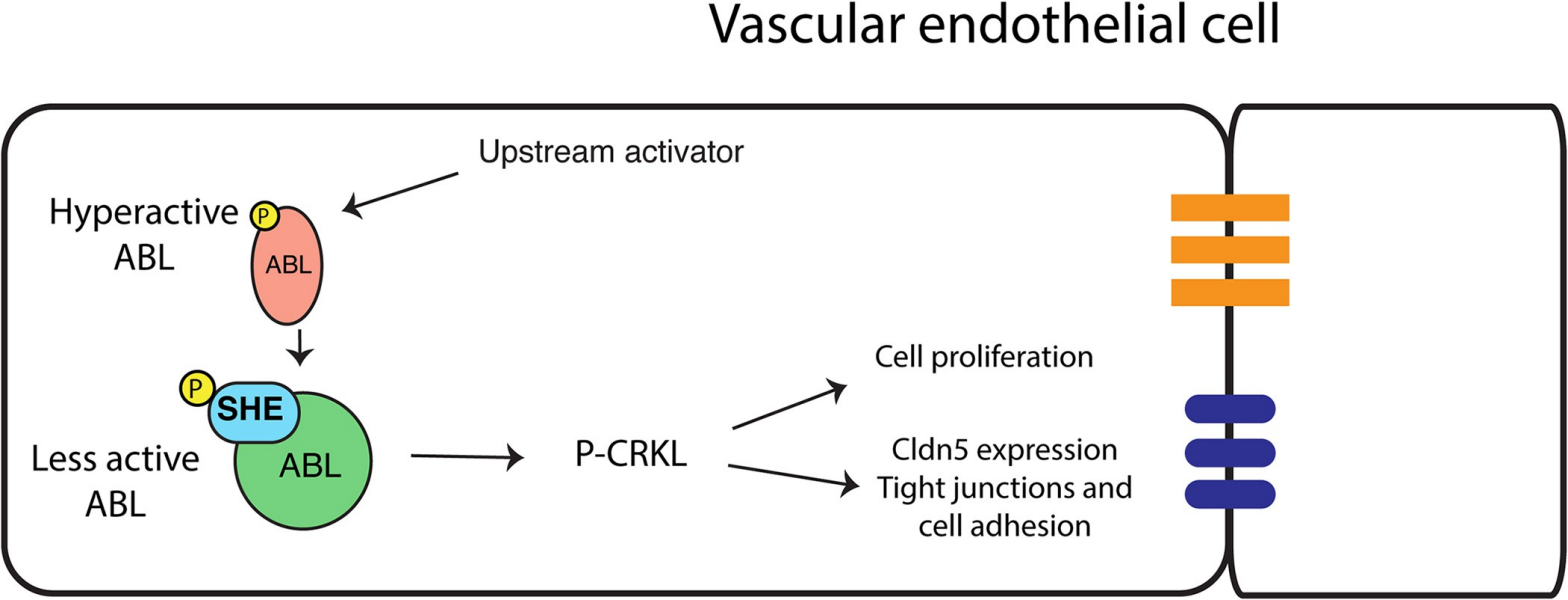
A proposed model for SHE and ABL signaling during vascular tubulogenesis. Activated ABL promotes enlarged vascular lumen through a downstream effector P-CRKL which increases endothelial cell proliferation and increases Cldn5 expression, thus affecting tight junctions and cell adhesion. Activated ABL phosphorylates SHE, which then interacts with ABL to dampen its activity resulting in the lumen of appropriate size.

Our results demonstrate that She functions as a negative regulator of blood vessel size. Loss of function *she* zebrafish mutants exhibit enlarged DA and have more arterial cells, while vascular endothelial *she* overexpression resulted in a reduced size DA with fewer cells. Enlarged DA size also correlated with an increase in vascular lumen size. Inhibition of Abl signaling resulted in a similar reduction in DA size and cell number in wild-type embryos and reversed DA enlargement observed in *she* mutants, suggesting that She functions as a negative regulator of Abl signaling. A previous study demonstrated a direct interaction between She and Abl kinase in a yeast two hybrid assay [24]. Abl binding partners are often also substrates and/or activators of Abl kinase. Based on our data, we hypothesize that *she* functions as an adaptor protein to inhibit Abl activity. According to this model, Abl phosphorylates She, which then acts as a negative regulator to reduce Abl activity (Fig 9). This type of feedback inhibition would allow for more accurate and sensitive regulation of vascular tube and lumen size and could be modulated by She expression or its phosphorylation level.

In support of this model, zebrafish She has four YXXP motifs that correspond to predicted Abl tyrosine phosphorylation sites [34], all of which are conserved in human and mouse. She mutant variant YXXP -> FXXP with substitutions of tyrosines to phenylalanines failed to rescue the *she* mutant phenotype, demonstrating the importance of all four consensus phosphorylation sites for She function. Notably, these ABL phosphorylation sites are located outside of the SH2 domain, which is consistent with our results showing that the She protein lacking the SH2 domain is sufficient to rescue *she* mutant embryos. These data strongly suggest that Abl kinase phosphorylates She on these consensus sites, which is then critical for She activity.

Although the role of Abl signaling in normal vascular tubulogenesis has not been previously known, recent work has established the role of ABL signaling during pathogenesis of venous malformations (VM). In a VM cell culture model which overexpresses activating Tie2 receptor with Tie2-L914F mutation, enlarged vascular tubes and increased phosphorylation of c-ABL was observed [23]. Inhibition of ABL signaling reduced vascular lumen size and caused VM regression. Our data suggest that ABL signaling may play a similar role during embryonic tubulogenesis by promoting enlargement of vascular lumen, while the upstream initiating signal remains unclear.

Abl kinase is known to signal through multiple cytoplasmic effector proteins in different cellular contexts. In endothelial cells, Abl can enhance Angiopoietin-mediated activation of the AKT and ERK signaling pathways [20]. It is also known to promote Rac1 activation and cell-cell adhesion by phosphorylating Crk and Crkl proteins, which are considered specific substrates of Abl kinase [15,35,36]. Our results show that Crkl phosphorylation is increased in She knockdown HUVEC cells, which suggests that Crkl functions as a downstream effector of She-Abl signaling in vascular tubulogenesis.

While our results strongly suggest that She functions in the Abl signaling pathway, it is important to note that the chemical inhibitors may also inhibit additional signaling pathways, and their specificity in vivo has not been fully determined. Therefore, we cannot exclude the possibility that the DA size decrease in GNF7 and dasatinib treated embryos may be caused by inhibition of other signaling pathways. Obtaining additional genetic evidence will be important to fully validate the role of Abl signaling in vascular tubulogenesis.

Rho GTPases (Rac1, Cdc42 and RhoA) have been previously implicated as key regulators of actomyosin network during vascular lumen formation [1–5]. Rac1 and Cdc42 stimulate cytoskeletal remodeling to establish cell polarity including localization and stabilization of cell junctions to regulate endothelial tubulogenesis, while RhoA has an opposing activity [3,8,37]. Abl signaling has been implicated in regulating Rac1 activity in different non-endothelial cell types [13–16], therefore it is likely that She and Abl signaling regulate vascular lumen size by modulating the activity of Cdc42 and Rac1 or other GTPases. In support of this model, our data demonstrate that phosphorylation of PAK4, a downstream effector of Cdc42 [7,33], is increased in *SHE* knockdown endothelial cells.

In addition to regulating cytoskeletal remodeling, Abl signaling is known to promote cell proliferation in different cell types, including smooth muscle cells and fibroblasts [18,19]. Our data argue that Abl signaling also promotes vascular endothelial cell proliferation in the DA, while She acts as an inhibitor of this process. It is quite likely that downstream effectors affecting cell proliferation and cytoskeletal rearrangements are different, which needs to be investigated further in the context of vascular tubulogenesis.

Intriguingly, *she* expression is primarily enriched in the arterial vasculature, including the DA [25]. *she* mutants had little if any change in the PCV diameter, while the DA diameter was enlarged as early as 28 hpf. Abl inhibition did not cause a significant change in the PCV diameter, while the DA was reduced. This suggests that Abl activity primarily regulates size of the arterial vasculature. Although vascular diameter in older embryos and adults is regulated by smooth muscle contraction, there is no smooth muscle cell coverage yet at 28 hpf. Therefore, DA enlargement is unlikely to be caused by defects in smooth muscle cells. Our results show that *cldn5a* expression, which encodes zebrafish homologs of Claudin 5, a major component of the tight junctions, is upregulated in *she* mutants. It is possible that She-Abl signaling directly represses *cldn5a* expression to ensure an optimal amount of Cldn5a protein. Alternatively, *cldn5a* expression may be upregulated as a secondary consequence of other changes in cell shape, junctions, or polarity. *cldn5a* mutants display reduction in the DA lumen size [32]. Our results show that downregulation of *cldn5a* expression reverses the phenotype of enlarged DA observed in *she* mutant embryos, suggesting the DA enlargement is mediated at least partially by an increase in *cldn5a* expression. In addition to mediating vascular permeability, Cldn5 has been implicated in cell motility and proliferation of endothelial and tumor cells [32,38,39]. It is possible that increased cell proliferation in the DA may be a direct or indirect consequence of upregulated *cldn5a* expression. Our results show that Cldn5a knockdown reduced the number of endothelial cells in the DA, thus supporting this model. Alternatively, increase in *cldn5a* expression may be independent of She effect on cell proliferation.

The SHE protein sequence is highly conserved between zebrafish and other vertebrates. Intriguingly, siRNA knockdown of human SHE in HUVECs resulted in a similar increase in vascular tube diameter. There is no blood flow or smooth muscle / pericyte cells in this 3D collagen assay, therefore the observed tube enlargement must be cell-autonomous to vascular endothelial cells. Endothelial-specific expression of She rescued the *she* loss-of-function phenotype in zebrafish embryos, further supporting cell autonomous role for SHE.

In summary, our results demonstrate novel roles for SHE and ABL signaling in regulating vascular tube and lumen size. These findings will contribute to our understanding of molecular mechanisms that regulate vascular tubulogenesis and eventually may facilitate development of novel therapeutic approaches for pathological disorders, such as coronary artery ectasia or venous malformations which display ectatic vessels with enlarged lumens.

## Materials and methods

### Ethics statement

All zebrafish work was performed after approval of University of South Florida and Cincinnati Children’s Hospital Medical Center Institutional Animal Care and Use Committees.

### Zebrafish lines

#### Generation of *she*^*ci26*^ line

TALEN genome editing was used to create the *she*^*ci26*^ allele. TALENs were designed using the TAL Effector Nucleotide Targeter software (https://tale-nt.cac.cornell.edu/) [40]. The TALEN target sequence TGTGCGTATTCGTCATGGCgaagtggtttaaagaTTTTCCAACTAGTTTGAAGA is located in exon 1 of the *she* gene. The lowercase letters represent the spacer sequence, which contains MseI restriction site TTAA. TALENs were assembled using Golden Gate assembly [41], and mRNA for the left and right arms was synthesized using the T3 mMessage mMachine kit (ThermoFisher). 100 pg of each mRNA was injected into one-cell stage wild type embryos. she_F1 and she_R1 primers were used to test TALEN efficacy and identify *she*^*ci26*^ adult carriers (primer sequences listed in S1 Table). These primers amplify a 333 bp product, which can then be digested with MseI.

#### Generation of *she*^*ci30*^ line

CRISPR/Cas9 editing was used to create the *she*^*ci30*^ allele. Guide RNAs were designed using ChopChop *(*http://chopchop.cbu.uib.no). Two separate guide RNA templates were generated by PCR as described [42], using she_gRNA1, she_gRNA2 and the common she_gRNA_R1 primer (S1 Table). gRNAs were synthesized using the MEGAshortscript T7 kit (Life Technologies). The two gRNAs were injected together with Cas9-NLS protein (New England Biolabs) into one-cell stage embryos. The injected animals were outcrossed and PCR was performed on pooled embryos to identify carriers of *she* mutations, using She_gRNA left and She_gRNA right primers (S1 Table). These primers span the large deletion in *she*^*ci30*^, and thus generate an approximately 200 bp PCR product only from the mutant allele. To identify the wild type allele, a separate PCR reaction was performed using She_gRNA midR reverse primer (S1 Table). This primer is located within the deleted region, thus will amplify a 234 bp product only from the wild type allele.

#### Generation of *fli1*:*she-2A-mCherry* and *hsp70*:*she-2A-mCherry* lines and DNA overexpression analysis

To create full length over-expression constructs, we amplified the *she* full length coding sequence from wild type cDNA using she_attB_F1 and she_attB_R3 primers (S1 Table) for Gateway cloning (Invitrogen). The PCR product was recombined via BP reaction into pDONR221. To make the *fli1*:*she-2A-mCherry* construct, the resulting middle entry vector (pDONR221-she) was then recombined via LR reaction with p5E-fli1ep and p3E-2AmCherry into the destination vector pDestTol2pA2 [43]. To make the hsp70-she-2A-mCherry construct, pDONR221-she, p5E-hsp70, and p3E-2A-mCherry were recombined via LR reaction into the destination vector pDestTol2pA; cryaa:dsRed. Induction of the *hsp70*:*she-2A-mCherry* transgene was confirmed by ubiquitous mCherry fluorescence, while expression of *fli1*:*she-2A-mCherry* was observed in vascular endothelial cells (S5 Fig).

To analyze mosaic overexpression, embryos were injected with 30 ng of *fli1*:*she-2A-mCherry* DNA construct and 225 ng of Tol2 mRNA mixture. Embryos with endothelial mCherry expression were chosen for confocal imaging and further analysis. mCherry positive and negative segments of the DA (1 segment per embryo) were chosen for measurements in Fiji software. 3 measurements were performed for each segment analyzed, and measurements were then averaged to obtain a single data point used for further calculations.

To create She deletion constructs, we amplified portions of *she* from wild type cDNA.

To generate sheΔSH2 which contains SH2 domain deletion, she_attB_F1 and she_deltaSH2_attB_R primers were used. To generate sheSH2 construct which contains SH2 domain only, sheSH2_attB_F and she_attB_R3 primers were used (S1 Table). Each product was recombined via BP reaction into pDONR221. The resulting middle entry vectors were each then recombined via LR reaction with p5E-fli1ep and p3E-2AmCherry into the destination vector pDestTol2pA2. All constructs were confirmed by sequencing. All final constructs were injected with Tol2 mRNA into one-cell stage embryos derived from a cross of *she*^*ci26*^ heterozygous animals. Injected embryos were raised to adulthood and outcrossed to produce embryos to screen for germline integration. Transgenic lines were identified based on vascular endothelial mCherry expression.

To create the *she-FXXP* construct, we performed sequential site-directed mutagenesis on each YXXP site starting with the pDONR221-she construct, using the QuikChange II Site-directed Mutagenesis Kit (Agilent). Each construct was fully sequenced before proceeding with the next round of mutagenesis. The primer pairs sheYXXP1-P4 used for mutagenesis of each site are listed in S1 Table. This construct was then combined via LR reaction with the p5E-fli1ep and p3E-2A-mCherry into the pDestTol2pA2 destination vector, then injected as described above. All *she* lines were maintained in *kdrl*:*GFP*^*s843*^ background [44].

#### Other lines

The following lines were used in the study: *kdrl*:*GFP*^*s843*^ [44], *Tg(kdrl*:*NLS-mCherry)*^*is4Tg*^ [45], *Tg(fli1*:*nGFP)*^*y7Tg*^ [46].

### Heat-shock induction

Embryos at the indicated stages were placed in 96 well PCR plates in embryo media with 5–10 embryos per well. To induce transgene expression, embryos were incubated at 37°C for one hour in a thermocycler. Following heat shock, embryos were transferred from PCR tubes to fresh media in petri dishes and sorted for dsRed expression in the lens or mCherry fluorescence in the body to indicate presence of the transgene. For DA diameter analysis, embryos were genotyped following confocal imaging and analysis.

### Abl inhibitor treatments

Dasatinib (Selleckchem) and GNF-7 (Selleckchem) were resuspended in DMSO to a concentration of 10 mM. Stocks were further diluted into embryo water to the appropriate working concentrations as noted. DMSO was diluted 1:1,000 or 1:10,000 in embryo water as a control. Pools of 20 dechorionated *kdrl*:*GFP* or *she*-/-; *kdrl*:*GFP*^*s843*^ embryos were placed in 1 ml of diluted inhibitor or diluted DMSO in glass vials starting at 6 hpf. Embryos were placed on a nutator in the dark until 24–28 hpf, when they were imaged for analysis.

### In situ hybridization and hybridization chain reaction analysis

Chromogenic whole mount in situ hybridization was performed as described [47]. *she* DIG-labeled antisense mRNA probe was synthesized as previously described [48]. To make ISH probe for *cldn5a*, 897 bp fragment corresponding to the coding sequence of *cldn5a* was amplified by PCR from zebrafish 24 hpf embryonic cDNA. The PCR product was subcloned into pCRII-TOPO using TOPO TA cloning kit (ThermoFisher). The construct was linearized using XhoI restriction endonuclease (ThermoFisher), and antisense DIG-labeled RNA was synthesized using SP6 RNA polymerase (Promega).

Fluorescent in situ hybridization using hybridization chain reaction (HCR) for *cldn5a* was performed as previously described [49,50]. Embryos were obtained from an incross of *she+/-; kdrl*:*GFP* parents, imaged using 10x or 20x objective on a Nikon A1R or Nikon Eclipse confocal microscope and subsequently genotyped. To measure fluorescence intensity of *cldn5a* staining, a small rectangular area which included a portion of the DA was selected using Fiji (Image J). Three measurements at different locations of the DA (using the same size rectangular) in the trunk region were performed for each embryo. In addition, a similar size area was selected in the *cldn5a* expression domain within the neural tube (for normalization) and in the embryo region which did not show any specific *cldn5a* expression (for background subtraction). Integrated density was calculated for each area. Subsequently, background subtraction was performed by subtracting integrated density values obtained for the area which did not show specific *cldn5a* expression. All DA expression values were then normalized by dividing them over the neural tube expression of the same embryo. This procedure reduced variation due to staining intensity variability between different embryos. *cldn5a* expression in the neural tube was not expected to be affected, therefore it served as a reference point. Student’s t-test was used to calculate statistical significance between normalized integrated density values of *cldn5a* expression in the DA of wild-type and *she* mutant sibling embryos. All measurements were performed blindly without a prior knowledge of embryo genotypes.

A similar approach was used to measure *cldn5b* intensity. HCR probes and fluorescent 647nm hairpins were obtained from Molecular Instruments Inc (Los Angeles, CA). Because *cldn5b* only stains DA but not the neural tube, normalization was not performed in this case. All measurement values per embryo (3 per embryo at different locations of the DA) were averaged, and single datapoints for each embryo were used for statistical analysis of *cldn5a* and *cldn5b* expression.

HCR for *pdgfrb* expression in *she; kdrl*:*GFP* embryos was performed under a similar protocol. *pdgfrb* probe was obtained from Molecular Instruments and used with 594nm hairpins. Fiji / Image J was used to measure the mean intensity of *pdgfrb* expression within the DA region that spans approximately 4 ISVs in each embryo. Data were combined from 3 separate replicate experiments. Because the overall staining intensity varied between separate experiments, relative fluorescence intensity was normalized within each experiment. For this purpose, mean *pdgfrb* fluorescence intensity values were calculated for wild-type embryos within each experiment. Then the measured intensity values for each wild-type or mutant embryo were divided over the same mean wt intensity value calculated for each experiment. The resulting relative expression values were used for the graph and statistical calculations.

### Morpholino injection

A previously validated *cldn5a* MO [32](GeneTools Inc, sequence AGGCCATCGCTTTCTTTTCCCACTC) was injected into embryos at 1-cell stage obtained from a cross of *she+/-; kdrl*:*GFP* parents. Estimated 2 ng dose per embryo was used for full phenotype, and 1 ng as a subphenotypic dose. Embryos from injected and control uninjected groups were imaged at 2 dpf using confocal microscopy (Nikon Eclipse) and genotyped. DA lumen diameter was analyzed as described above. All analysis was performed blindly without knowing embryo genotypes.

To inhibit heart beat and blood flow, 4 ng of previously validated *tnnt2a* MO [27](GeneTools Inc, sequence CATGTTTGCTCTGATCTGACACGCA) was injected at the 1-cell stage into embryos from *she+/-; kdrl*:*GFP* incross. Confocal imaging was performed at 48 hpf, and embryos were subsequently genotyped. DA width was measured using Imaris 10.0 software at three different points (each point was selected at the midpoint of DA between two consecutive intersegmental vessels) in each embryo, and the mean values were calculated and used for statistical analysis.

### qPCR analysis using FACS-sorted endothelial cells

Wild-type and *she* mutant embryos were obtained from respective incrosses of *she+/+; fli1*:*she-2A-mCherry; kdrl*:*GFP* and *she-/-; fli1*:*she-2A-mCherry; kdrl*:*GFP* parent fish (which were siblings). 15–20 mCherry-negative embryos were obtained for each group at 24 hpf stage. Single-cell suspension was obtained as previously described [51]. FACS sorting for GFP-positive cells was performed at the CCHMC FACS Core facility. Total RNA was purified using RNA purification kit (Norgen Biotek). cDNA was synthesized at CCHMC Gene Expression Core facility. Quantitative real-time PCR (qRT- PCR) was carried out using PowerUp SYBR Green Master Mix (Applied Biosystems) in a StepOnePlus Real-Time PCR System. qPCR amplification for *cldn5a* and EF1α was performed using the following primers: cldn5a-F (GACAACGTGAAAGCGCGGG), cldn5a-R (AGGAGCAGCAGAGTATGCTTCCC), EF1a-F (TCACCCTGGGAGTGAAACAGC) and EF1a-R (ACTTGCAGGCGATGTGAGCAG). Fold changes were calculated using a relative standard curve methods and normalized to EF1a expression.

### Microscopy imaging

For brightfield imaging, *she*^*ci30*^ and *she*^*ci26*^ embryos were obtained by respective crosses of heterozygous parents. Live embryos were anesthetized in 0.016% Tricaine solution, mounted in 2% methylcellulose and imaged using Zeiss Axioimager (10x objective). Embryos were subsequently genotyped. For brightfield imaging of embryos after in situ hybridization, they were mounted in 0.6% low-melting-point agarose and imaged using 10x objective on a Nikon Eclipse compound microscope. In some cases, when the entire embryo did not fit in the field of view, separate images of the anterior and posterior portions of embryo were obtained and merged using Adobe Photoshop (Fig 1B–1E).

Fluorescent imaging of live embryos and fixed embryos after HCR was performed using Nikon confocal microscope (A1R or Eclipse). Embryos were mounted in 0.6% low melting-point-agarose. Nikon Elements AR Denoise.ai algorithm was used to remove noise from images with high background signal. Maximum intensity projections were obtained using Nikon Elements or Fiji (Image J) software. Image levels were adjusted using Adobe Photoshop 2021 to maximize brightness and contrast; in all cases similar adjustments in control and experimental embryos were performed.

### Measurements of DA and PCV diameter

To analyze the DA and PCV diameter of *she*^*ci26*^ mutants, embryos were obtained by a cross of heterozygous parents. Live embryos were anesthetized in Tricaine, mounted in 0.6% LMP agarose and imaged using Nikon A1 confocal microscope. Subsequently embryos were retrieved and genotyped. Measurements were performed in Fiji (Image J). Four measurements across the DA or PCV were taken in different areas of the mid-trunk for each embryo. Measurements were performed at the midpoint between two ISVs, every other ISV. The mean value was then calculated for each embryo, and this value was used in statistical analysis. Measurements were performed blindly without knowledge of embryo genotypes. DA diameter was measured similarly in embryos treated with Abl inhibitors. In the experiment where *she* mutant and control embryos were treated with GNF7 (Fig 5), 3 measurements within the DA in each embryo were performed, and subsequently mean value was calculated (embryos were imaged at higher magnification, and thus a smaller region of DA in the trunk was available for analysis). In the experiment where *she* and mutant embryos were injected with *cldn5a MO* (Fig 7I–7L), 5 measurements within the DA in each embryo were performed and used to calculated mean value (lower magnification imaging was used for this analysis covering a larger region of an embryo).

### Cell number analysis

To analyze arterial cell number in *she* mutants, embryos were obtained from an incross of *she+/-; kdrl*:*GFP; kdrl*:*NLS-mCherry* adults and imaged using confocal microscopy under a 20x objective at 72 hpf. Embryos were then extracted from the LMP agarose and genotyped. Nuclei cell counts analysis of the DA region was performed within a segment between 5 ISVs using Imaris 7.2 software. First, the region of interest was selected to limit the counts within the DA. An estimated diameter was calculated by measuring the size of selected cell nuclei within the Imaris. Then, the preliminary results were manually corrected to remove or add nuclei based on the confocal 3D image. Similar approach was used for cell counts in GNF7 treated embryos and DMSO controls using *kdrl*:*GFP; kdrl*:*NLS-mCherry* line.

To count cells in the DA in *she* mutants at 28 hpf, fixed embryos from *she+/-; kdrl*:*GFP; kdrl*:*NLS-mCherry* parents were imaged and subsequently genotyped. In this case, the cells were counted in a segment between 3 ISVs using Imaris 10.0 software, since embryos were imaged at higher magnification to aid in resolving individual nuclei.

To count arterial cells in She overexpressing embryos, double transgenic *fli1*:*she-2A-mCherry; fli1*:*NLS-GFP* embryos were obtained from a cross of separate transgenic parents. Confocal imaging at 28 hpf and 72 hpf and cell counts were performed in GFP channel as described above for 72 hpf analysis, except that the nuclei were counted in the region of 4 ISVs at 28 hpf stage.

### Cell proliferation analysis

Embryos were obtained through an incross of *she-/-; fli1*:*she-2A-mCherry; kdrl*:*GFP* adult zebrafish. mCherry positive and negative embryos were segregated by screening under a fluorescent microscope. The embryos were treated with BrdU (Millipore-Sigma, Cat No. B5002) in a 24-well plate (20 embryos per well in a 200μl of 15 mM BrdU solution in 15% DMSO) from 29 to 29.5 hpf. At 29.5 hpf, the embryos were allowed to recover in embryo water for 30 minutes and fixed afterwards at 30hpf. The embryos were fixed in BT-fix for 2 hours at room temperature (RT). Fixed embryos were walked with gradual changes into 100% methanol and kept overnight at -20°C. The next day, embryos were walked into PBT (PBS + 0.1% Tween 20). The embryos were washed with PBT for 3X15 mins. This was followed by incubation in 2N HCL for 1 hour at RT. The embryos were washed with PBT for 3X5 mins. The embryos were then placed in blocking buffer for 2.5 hours. This was followed by overnight incubation in primary anti-BrdU antibody (ab6326, abcam, 1:250 dilution). The embryos were washed the next day with PBT for 6X15 mins. This was followed by overnight incubation in the goat anti-rat Alexa Fluor 647 secondary antibody (A21247, ThermoFisher, 1:500). The embryos were washed with PBT 4X15 min and kept at 4°C overnight. The embryos were imaged the next day on a Nikon Eclipse confocal microscope with 20X water immersion objective. The entire trunk region was imaged in separate segments (3 images / embryo). The double labelled cells were counted using the Imaris software 9.1 (Bitplane). A region of interest was drawn to encompass only the DA. Separate spots were created for *kdrl*:*GFP* positive cells and anti-BrdU labeled cells, respectively. Co-localization was defined as spots by no more than 4μm. Colocalization and cell identity was confirmed by manual analysis of the image. The data were obtained from two independent experiments.

### Intersegmental vessel analysis

To count arterial and venous connections, *she*^*ci26+/*^*-; kdrl*:*GFP* adults were crossed to obtain a mixture of *she* homozygous, heterozygous and wild-type embryos, which were then mounted in 0.6% LMP agarose and imaged by confocal microscopy using a 10x objective at 72 hpf. Embryos were then extracted from the agarose and genotyped. Obtained images were analyzed blindly without knowing embryo genotype to determine arterial or venous connections of 10 consecutive pairs (20 ISVs per embryo.)

### Qtracker dot injection

The embryos were obtained by an incross between *she+/-; kdrl*:*GFP* parents. Approximately 4 to 5 nl of a 2 μM solution of Qtracker705 label (Qtracker 705 Vascular Labels, Thermo Fisher Scientific) was microinjected into the common cardinal vein of each embryo adjacent to the sinus venous at 2 dpf. The embryos were imaged by confocal microscopy using 20x objective one hour post injection and genotyped afterwards. For calculating the area density of DA (area per unit length of DA), the area and length of a specific region of DA were measured for each embryo using Imaris 10.0 software. The area was divided over the length to obtain the value corresponding to the DA width. Each value was then divided over the average DA width in wt embryos to obtain the final relative DA size value.

### Abl CRISPR / Cas9 injection

gRNAs targeting the exons of both *abl1* and *abl2* genes were designed using CHOPCHOP website [52] (https://chopchop.cbu.uib.no/) and were synthesized from Synthego Inc, using Synthego modified EZ Scaffold option. The sequence of gRNAs is shown in S1 Table. All six gRNAs were added in equal amounts to the final concentration of 6 μM of total gRNA and 4 μM Cas9 protein and 1x Cas9 buffer from New England Biolabs (Cat No. M0646T). The reaction mixture was incubated at 37°C for 5 min and allowed to cool down to room temperature before performing microinjections. Approximately 2 nl of Cas9/gRNA mixture was injected into a blastomere of each *kdrl*:*GFP* embryo.

### EC tube formation assays in 3D collagen matrices

Human umbilical vein endothelial cells (ECs) were grown and used from passages 3–6 as previously described [53]. Human ECs were seeded in 3D collagen matrices in the presence of a combination of recombinant growth factors, stem cell factor (SCF), interleukin-3 (IL-3), stromal-derived factor-1 alpha (SDF-1α), and fibroblast growth factor-2 (FGF-2), which were added to Medium 199 containing a 1:250 dilution of reduced serum supplement-2 [53], which has insulin as a component. The recombinant growth factors were obtained from R&D Systems and were added at the following concentrations: SCF, IL-3, and SDF-1α at 40 ng/ml and FGF-2 at 50 ng/ml. The details of the serum-free defined assay system set-up have been described previously [54,55]. siRNA suppression of ECs was performed as described [53], using two rounds of transfection using either a control siRNA or an siRNA directed to She. Silencer select siRNAs (Invitrogen) were utilized for these experiments with a control siRNA (Negative control #2- catalog number 4390896) and a She siRNA (catalog number s43069). After siRNA treatment, ECs were seeded in 3D collagen matrices in the presence of the recombinant growth factors, and after 48 or 72 hr, cultures were either fixed with 3% glutaraldehyde in phosphate-buffered saline or collagen gels were plucked and lysates were prepared using SDS-PAGE Laemmli 1.5X sample buffer. Western blots were run and probed with antibodies directed to phospho-Pak2, phospho-Pak4, phospho-Erk, phospho-Crkl (Tyr207, Cat No. 3181S), total Pak2, total Pak4, total Erk and total Crkl (Cat No. 38710S) (Cell Signaling Technology). The anti-She antibody was from Novus and the anti-actin antibody was from Calbiochem. Fixed cultures were stained with 0.1% toluidine blue, and cultures were photographed using an Inverted Olympus microscope, and tube area was traced and quantitated using Metamorph software. Representative experiments were analyzed, and the data was obtained from triplicate wells while vessel tube area was measured from n = 6 or 7 fields. Statistical analysis utilized a Student’s t-test.

### Original numerical data

All numerical data used for graphs and summary statistics is listed in S2 Table.

## Supporting information

S1 Fig Diagrams and sequence chromatograms of *she*^*ci26*^ and ^*ci30*^ mutations. (A) *she*^*ci26*^ mutants have a 7 bp deletion. Alignment of wild-type and *she*^*ci26*^ mutant sequences is shown starting with the first coding ATG. (B) Genomic DNA sequence chromatogram of *she*^*ci30*^ mutants. (C) *she*^*ci30*^ mutants carry a 575 bp deletion and 24 bp insertion present between exons 3 and 4 within *she* gene.
(TIF)

S2 Fig Trunk intersegmental vessel analysis in *she-/-* and wt sibling embryos. (A,B) Confocal images of the trunk region of live wt and *she-/-* sibling embryos in *kdrl*:*GFP* background at 72 hpf. Selected arterial (aISV) and venous (vISV) intersegmental vessels are shown. (C) The number of aISV and vISV was counted in a selected region in wt and *she* mutant embryos. Embryos were genotyped after the imaging. Data were combined from two replicate experiments, each shown in different color. The number of embryos analyzed is shown at the bottom of each bar. No significant difference between wt and *she* mutant embryos was observed (p>0.05, t-Student’s test).
(TIF)

S3 Fig Endothelial marker expression, analyzed by in situ hybridization, is unaffected in *she* mutants at 24 hpf. (A, B) *kdrl* is strongly expressed in the dorsal aorta (DA) and intersegmental vessels (ISVs) and weakly expressed in the posterior cardinal vein (PCV) of both wild-type sibling and *she -/-* mutant embryos. (C, D) *flt1* expression is restricted to the DA and ISVs of both wild-type and *she -/-* mutants. (E, F) *dab2* is strongly expressed in the PCV and lightly expressed in the DA of both wild-type and *she -/-* mutants. Trunk region is shown in all panels. Embryos were obtained from an incross of *she+/-; kdrl*:*GFP* parents and subsequently genotyped.
(TIF)

S4 Fig Blood flow does not affect *she* expression or *she* mutant phenotype. (A,B) In situ hybridization analysis for *she* expression at 28 hpf in *tnnt2* MO-injected embryos and uninjected controls. Trunk region is shown, anterior is to the left. Numbers in the lower right indicate embryos that showed normal expression pattern out of the total number of embryos in 3 replicate experiments. (C-H) DA size analysis at 28 hpf in wt *(she+/+)* and *she-/-* sibling embryos, injected with *tnnt2* MO, compared to uninjected controls. Embryos were obtained from cross of *she+/-* parents in *kdrl*:*GFP* background and subsequently genotyped. Note that DA diameter is greatly reduced in *tnnt2* MO-injected embryos compared to wild-type uninjected embryos, and increased in *she* mutants, injected with *tnnt2* MO compared to wild-type embryos injected with *tnnt2* MO. Data are combined from 3 replicate experiments, shown in different color. Mean±SD is shown. The number of embryos analyzed is shown at the bottom of each bar. The same wt+*tnnt*2 MO embryos were used for comparisons in (G) and (H). *p<0.05, ****p<0.0001, Student’s t-test.
(TIF)

S5 Fig Generation of wild-type and mutant vascular endothelial-specific *she* zebrafish lines. (A-C) Fluorescent microscopy image of *fli1*:*she-2A-mCherry; kdrl*:*GFP* embryo at 3 dpf. (D) A diagram of She deletion constructs.
(TIF)

S6 Fig Inducible She expression under a heat-shock promoter *hsp70* rescues circulation defects in *she* mutants. (A,B) Pericardial edema is observed in *she* mutant embryos at 4 dpf (A), while embryos positive for *hsp70*:*she-2A-mCherry* do not show pericardial edema and have normal blood circulation after heat-shock was performed at 3 dpf (B). Embryos were obtained from the cross of *hsp70*:*she-2A-mCherry; cryaa*:*dsRed +/-; she+/-* X *she+/-* parents in *kdrl*:*GFP* background. Embryos were sorted for dsRed in the lens (driven by the lens specific *cryaa* promoter which correlates with the presence of *hsp70*:*she-2A-mCherry* transgene) and mCherry in the body. Representative embryos with and without edema are shown for each group. (C-F) Dorsal aorta in *she* mutants is narrower or collapsed at 4 dpf, while heat-shock at 3 dpf restores normal DA size in *she-/-; hsp70*:*she-2A-mCherry* embryos. (G) Percentage of total embryos showing the pericardial edema phenotype at 4 dpf. Embryos were obtained by crossing *hsp70*:*she-2A-mCherry; cryaa*:*dsRed +/-; she+/-* X *she+/-* adults in *kdrl*:*GFP* background and subjected to heat-shock (HS) at 3 or 3.5 dpf. Embryos with dsRed in the lens (Tg+) or transgene negative controls (Tg-) were selected for the analysis. Tg- embryos were also subjected to heat shock at 3 or 3.5 dpf (both groups were combined). *p<0.05, ****p<0.0001, Fisher’s exact test, compared to Tg- embryos. (H) The diameter of the dorsal aorta at 4 dpf in wild-type siblings (includes heterozygous and wild-type embryos), *she* mutant embryos without the transgene, and *she-/-; hsp70*:*she-2A-mCherry* embryos heat-shocked at 3 or 3.5 dpf. 3 replicate experiments were performed except for HS 3.5 dpf where two experiments were performed; data points from each replicate are shown in different color. Total number of embryos (n) for each group is shown at the bottom of each column. **** adjusted p<0.0001, Šidak’s multiple comparisons test, one-way ANOVA analysis.
(TIF)

S7 Fig *pdfgrb* mRNA expression analysis by hybridization chain reaction (HCR) at 48 hpf. (A,B) *pdgfrb* (red) expression in the trunk region of wild-type sibling and *she* mutant embryos in *kdrl*:*GFP* background at 48 hpf. Fluorescence within the dorsal aorta region (boxed) was selected for quantification. (C) Quantification of relative *pdgfrb* mRNA expression. No significant difference was observed (NS, p>0.05, Student’s t-test). Data were combined from 3 replicate experiments, shown in different colors. Mean±SD is shown.
(TIF)

S8 Fig Expression of *abl1* and *abl2* in different cell types during zebrafish embryogenesis based on single-cell RNA seq expression atlas. Data were generated by Daniocell atlas https://daniocell.nichd.nih.gov/ [31].
(TIF)

S9 Fig CRISPR/ Cas9 knockdown of *abl1* and *abl2* function results in reduced DA size. (A) A diagram illustrating the targeting sites of sgRNAs against *abl1* and *abl2* genes. Each gRNA was more than 90% effective based on DNA sequencing analysis. (B-D) Analysis of DA size in *kdrl*:*GFP* embryos at 28 hpf. Note the reduced DA diameter in embryos injected with *abl1* and *2* gRNA mixture. Data from two replicate experiments are shown in different colors. Mean±SD is shown. **p<0.01, Student’s t-test.
(TIF)

S10 Fig Hybridization chain reaction (HCR) analysis of *cldn5b* mRNA expression at 24 hpf. (A,B) *cldn5b* (purple) and *kdrl*:*GFP* fluorescence in the trunk region of *she* mutant and wild-type sibling embryos. DA, dorsal aorta; PCV, posterior cardinal vein. *cldn5b* fluorescence is shown in A’,B’. (C) Quantification of *cldn5b* fluorescence in the DA. p = 0.17, Student’s t-test. Error bars show SEM. Data show combined results from two independent experiments.
(TIF)

S11 Fig Western blots for expression of SHE and phosphorylation of PAK4, PAK2, ERK and CRKL in HUVEC cells transfected with a control or SHE siRNA at 48 and 72 hr time-points after transfection. Replicate experiments for pPAK4 and pCRKL phosphorylation are shown in (B). Selected bands, which showed the greatest change, are displayed in the main Fig 8.
(TIF)

S1 Table Primer and gRNA sequences sequences used to generate *she* mutant and transgenic lines, *abl1 /2* F0 embryos and perform qPCR. (XLSX)

S2 Table Numerical original data used for graphs and summary statistics. Tab numbers in the Excel table match to the figure numbers of corresponding graphs.
(XLSX)

S1 Movie Blood flow in the tail region of wild-type zebrafish embryos. Wild-type siblings obtained from an incross of *she*^*ci26*^ heterozygous adults in *kdrl*:*GFP; gata1*:*dsRed* background were imaged at 4 dpf using confocal microscopy.
(MP4)

S2 Movie Blood flow in the tail region of *she* mutant embryos. Mutant embryos obtained from an incross of *she*^*ci26*^ heterozygous adults in *kdrl*:*GFP; gata1*:*dsRed* background were imaged at 4 dpf using confocal microscopy.
(MP4)
