# Supplementary figures and images for: SH2 domain protein E and ABL signaling regulate blood vessel size
Source: PLoS Genet. 2024 Jan 8;20(1):e1010851. doi: 10.1371/journal.pgen.1010851 (PMC10798624; doi:10.1371/journal.pgen.1010851)

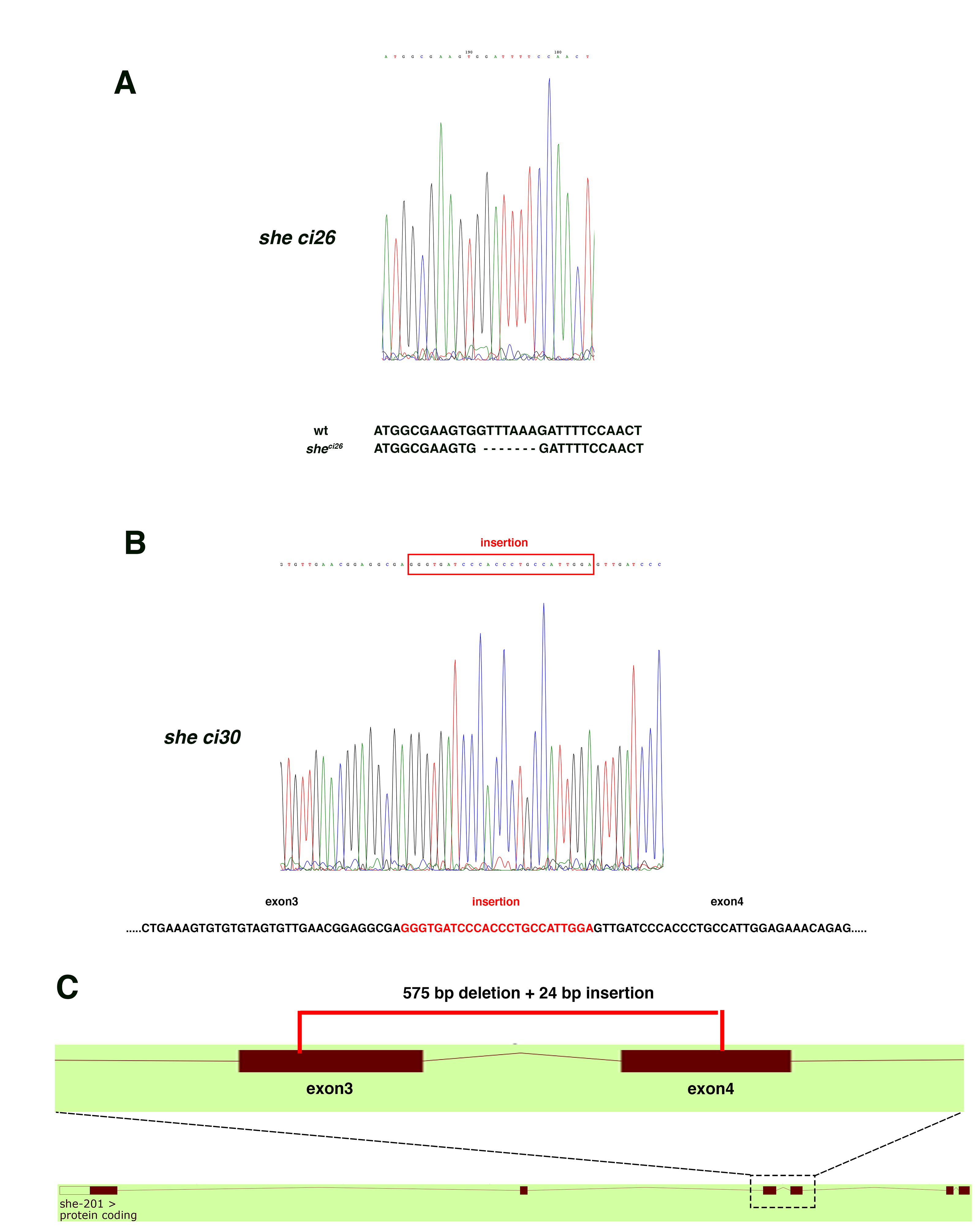

Supplement: S1 Fig — (A) sheci26 mutants have a 7 bp deletion. Alignment of wild-type and sheci26 mutant sequences is shown starting with the first coding ATG. (B) Genomic DNA sequence chromatogram of sheci30 mutants. (C) sheci30 mutants carry a 575 bp deletion and 24 bp insertion present between exons 3 and 4 within she gene. (TIF) [file pgen.1010851.s001.tif]

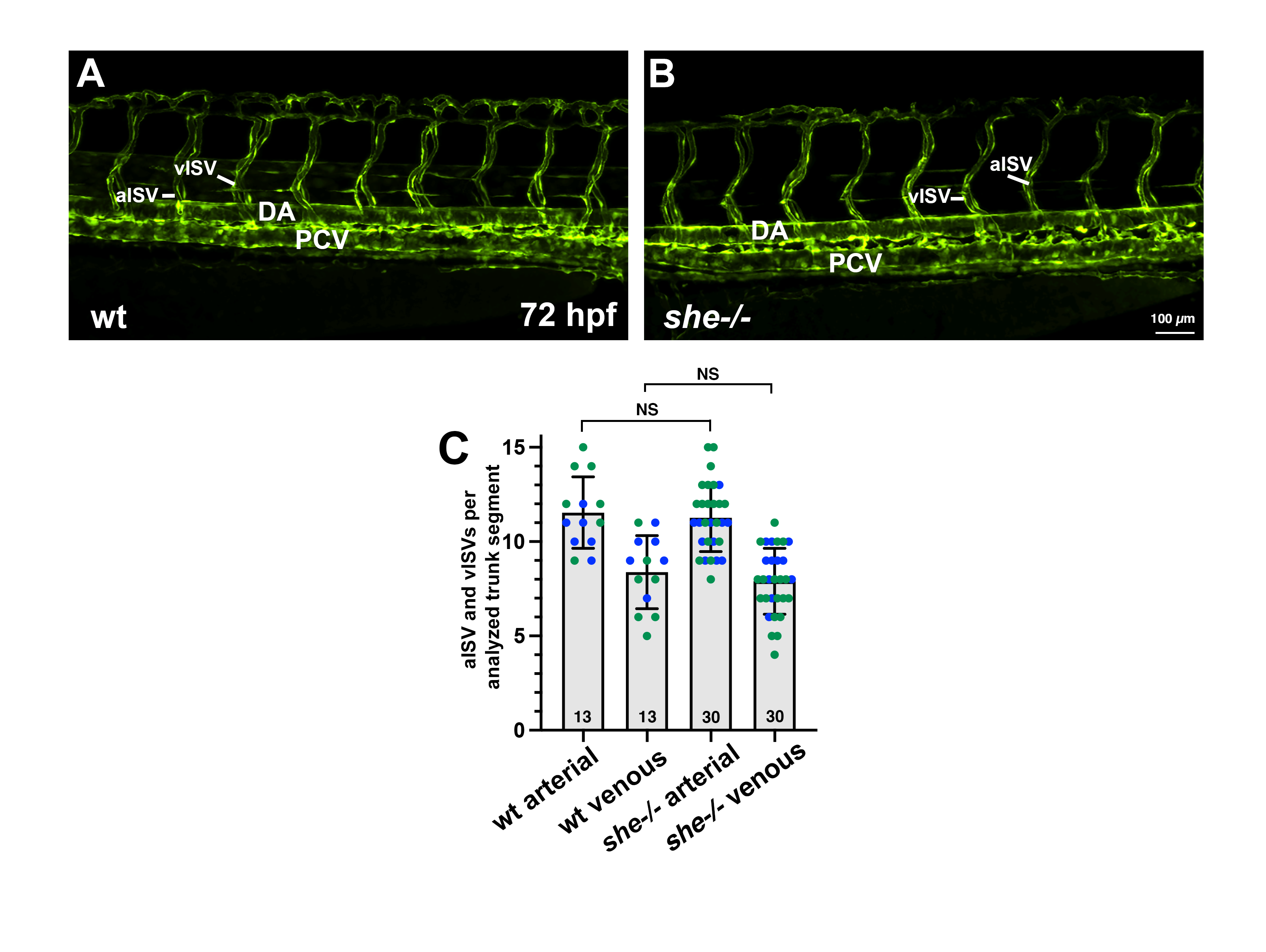

Supplement: S2 Fig — (A,B) Confocal images of the trunk region of live wt and she-/- sibling embryos in kdrl:GFP background at 72 hpf. Selected arterial (aISV) and venous (vISV) intersegmental vessels are shown. (C) The number of aISV and vISV was counted in a selected region in wt and she mutant embryos. Embryos were genotyped after the imaging. Data were combined from two replicate experiments, each shown in different color. The number of embryos analyzed is shown at the bottom of each bar. No significant difference between wt and she mutant embryos was observed (p>0.05, t-Student’s test). (TIF) [file pgen.1010851.s002.tif]

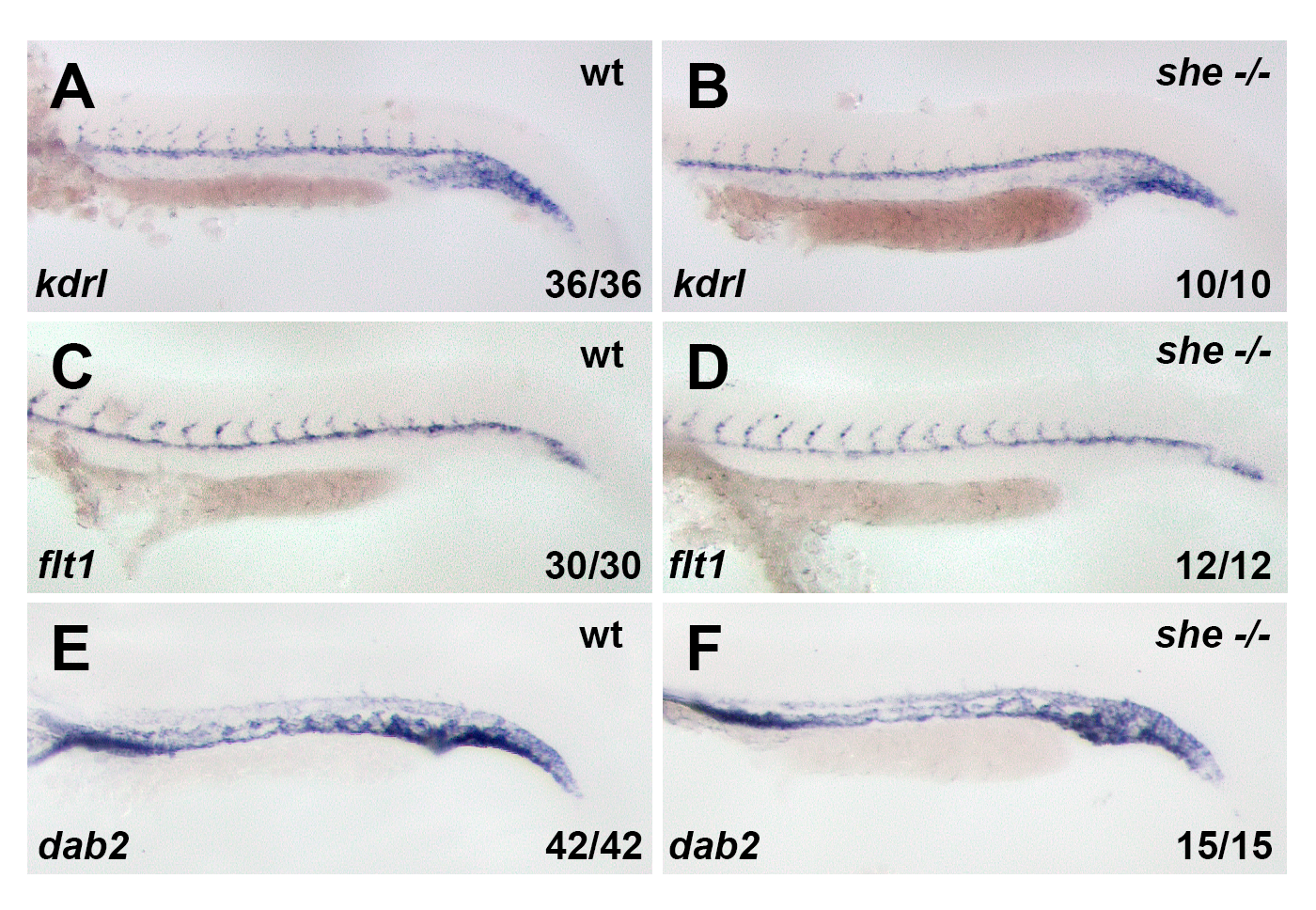

Supplement: S3 Fig — (A, B) kdrl is strongly expressed in the dorsal aorta (DA) and intersegmental vessels (ISVs) and weakly expressed in the posterior cardinal vein (PCV) of both wild-type sibling and she -/- mutant embryos. (C, D) flt1 expression is restricted to the DA and ISVs of both wild-type and she -/- mutants. (E, F) dab2 is strongly expressed in the PCV and lightly expressed in the DA of both wild-type and she -/- mutants. Trunk region is shown in all panels. Embryos were obtained from an incross of she+/-; kdrl:GFP parents and subsequently genotyped. (TIF) [file pgen.1010851.s003.tif]

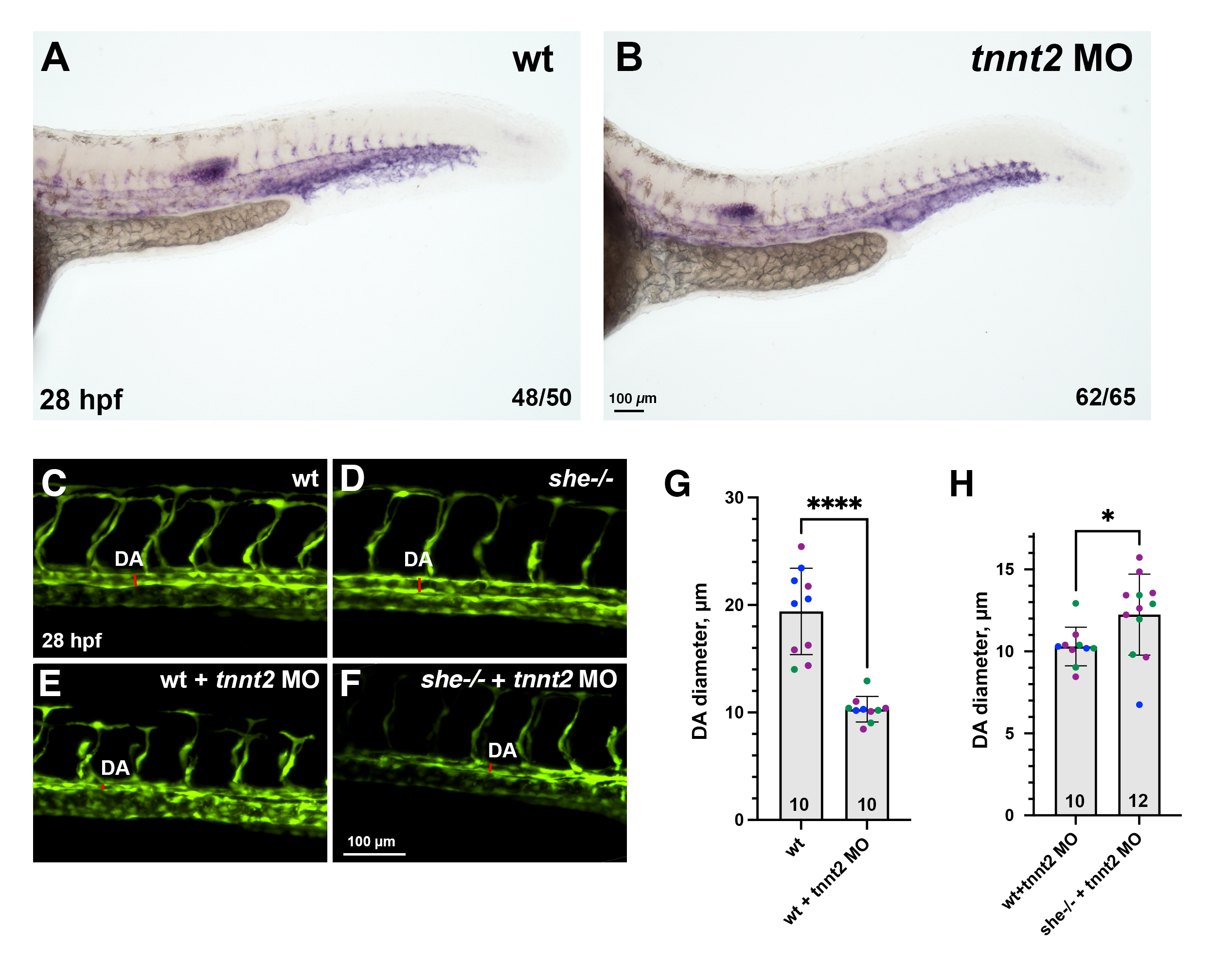

Supplement: S4 Fig — (A,B) In situ hybridization analysis for she expression at 28 hpf in tnnt2 MO-injected embryos and uninjected controls. Trunk region is shown, anterior is to the left. Numbers in the lower right indicate embryos that showed normal expression pattern out of the total number of embryos in 3 replicate experiments. (C-H) DA size analysis at 28 hpf in wt (she+/+) and she-/- sibling embryos, injected with tnnt2 MO, compared to uninjected controls. Embryos were obtained from cross of she+/- parents in kdrl:GFP background and subsequently genotyped. Note that DA diameter is greatly reduced in tnnt2 MO-injected embryos compared to wild-type uninjected embryos, and increased in she mutants, injected with tnnt2 MO compared to wild-type embryos injected with tnnt2 MO. Data are combined from 3 replicate experiments, shown in different color. Mean±SD is shown. The number of embryos analyzed is shown at the bottom of each bar. The same wt+tnnt2 MO embryos were used for comparisons in (G) and (H). *p<0.05, ****p<0.0001, Student’s t-test. (TIF) [file pgen.1010851.s004.tif]

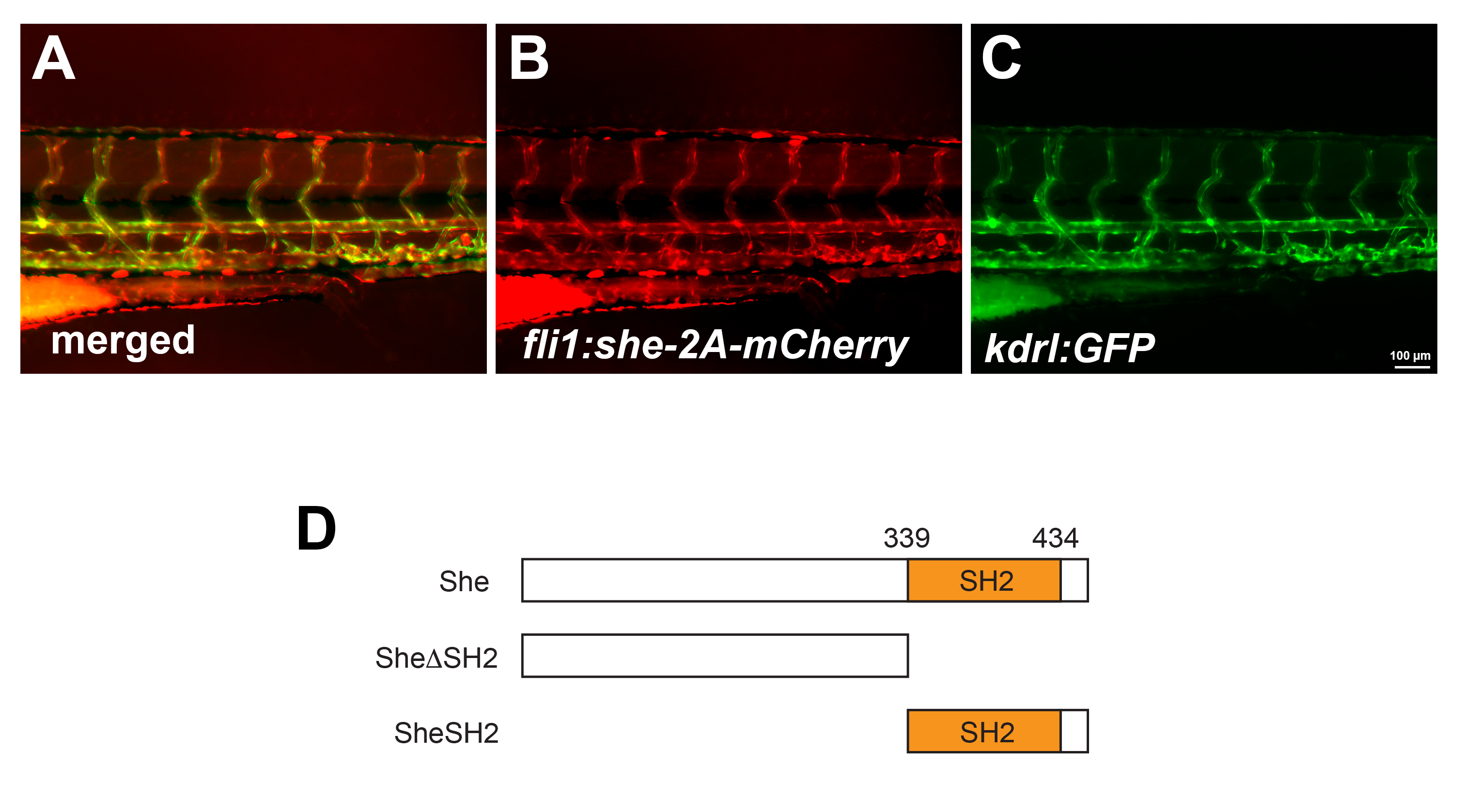

Supplement: S5 Fig — (A-C) Fluorescent microscopy image of fli1:she-2A-mCherry; kdrl:GFP embryo at 3 dpf. (D) A diagram of She deletion constructs. (TIF) [file pgen.1010851.s005.tif]

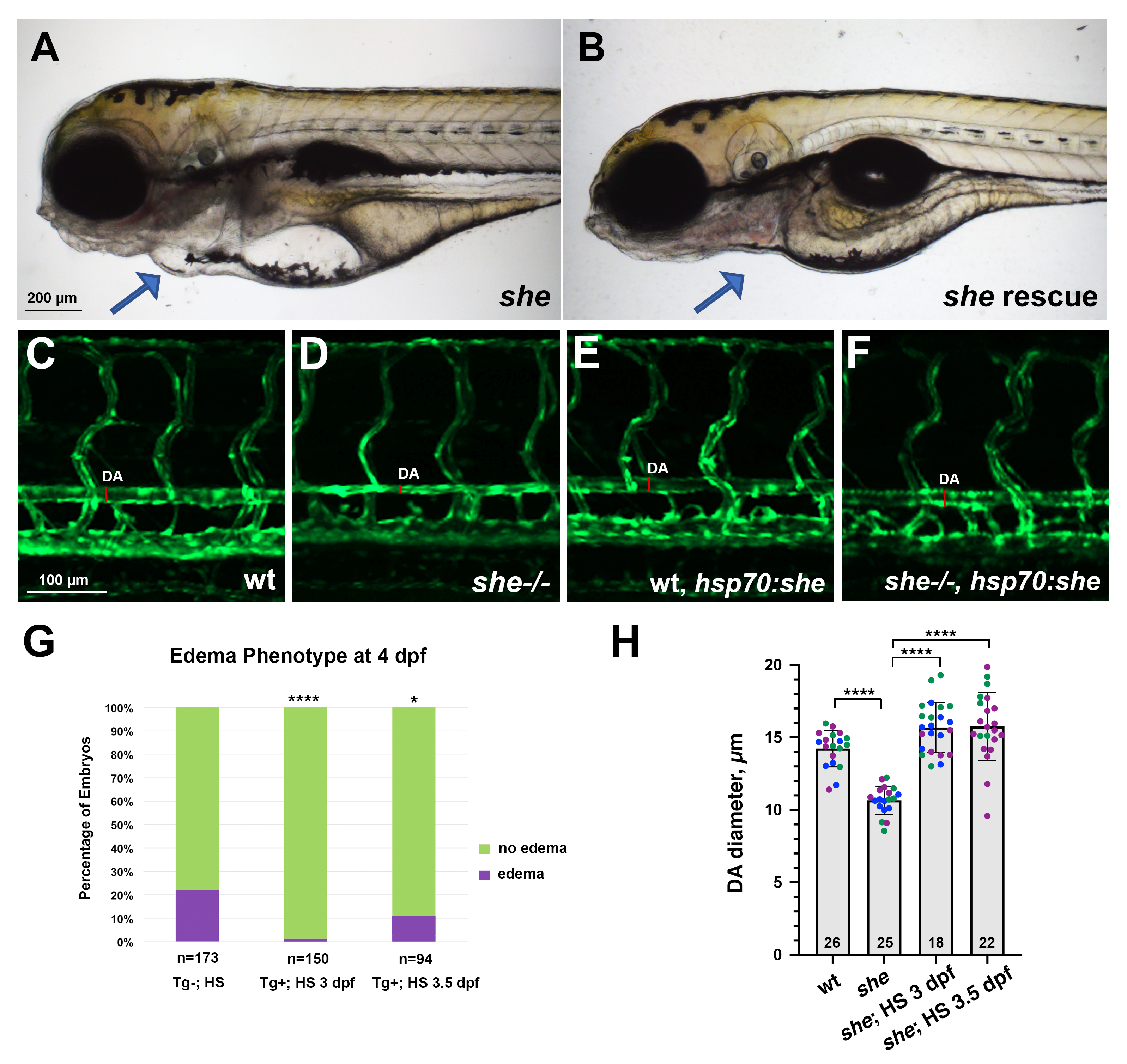

Supplement: S6 Fig — (A,B) Pericardial edema is observed in she mutant embryos at 4 dpf (A), while embryos positive for hsp70:she-2A-mCherry do not show pericardial edema and have normal blood circulation after heat-shock was performed at 3 dpf (B). Embryos were obtained from the cross of hsp70:she-2A-mCherry; cryaa:dsRed +/-; she+/- X she+/- parents in kdrl:GFP background. Embryos were sorted for dsRed in the lens (driven by the lens specific cryaa promoter which correlates with the presence of hsp70:she-2A-mCherry transgene) and mCherry in the body. Representative embryos with and without edema are shown for each group. (C-F) Dorsal aorta in she mutants is narrower or collapsed at 4 dpf, while heat-shock at 3 dpf restores normal DA size in she-/-; hsp70:she-2A-mCherry embryos. (G) Percentage of total embryos showing the pericardial edema phenotype at 4 dpf. Embryos were obtained by crossing hsp70:she-2A-mCherry; cryaa:dsRed +/-; she+/- X she+/- adults in kdrl:GFP background and subjected to heat-shock (HS) at 3 or 3.5 dpf. Embryos with dsRed in the lens (Tg+) or transgene negative controls (Tg-) were selected for the analysis. Tg- embryos were also subjected to heat shock at 3 or 3.5 dpf (both groups were combined). *p<0.05, ****p<0.0001, Fisher’s exact test, compared to Tg- embryos. (H) The diameter of the dorsal aorta at 4 dpf in wild-type siblings (includes heterozygous and wild-type embryos), she mutant embryos without the transgene, and she-/-; hsp70:she-2A-mCherry embryos heat-shocked at 3 or 3.5 dpf. 3 replicate experiments were performed except for HS 3.5 dpf where two experiments were performed; data points from each replicate are shown in different color. Total number of embryos (n) for each group is shown at the bottom of each column. **** adjusted p<0.0001, Šidak’s multiple comparisons test, one-way ANOVA analysis. (TIF) [file pgen.1010851.s006.tif]

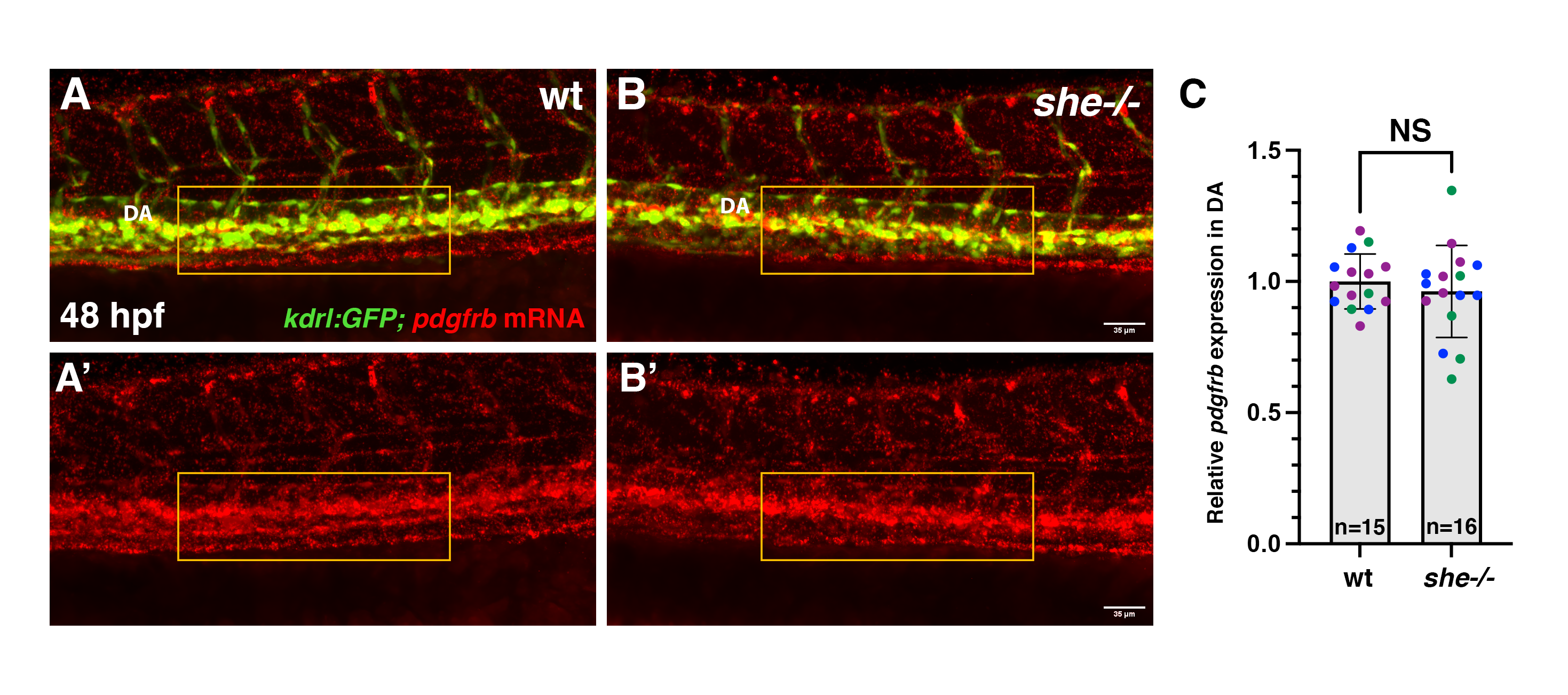

Supplement: S7 Fig — (A,B) pdgfrb (red) expression in the trunk region of wild-type sibling and she mutant embryos in kdrl:GFP background at 48 hpf. Fluorescence within the dorsal aorta region (boxed) was selected for quantification. (C) Quantification of relative pdgfrb mRNA expression. No significant difference was observed (NS, p>0.05, Student’s t-test). Data were combined from 3 replicate experiments, shown in different colors. Mean±SD is shown. (TIF) [file pgen.1010851.s007.tif]

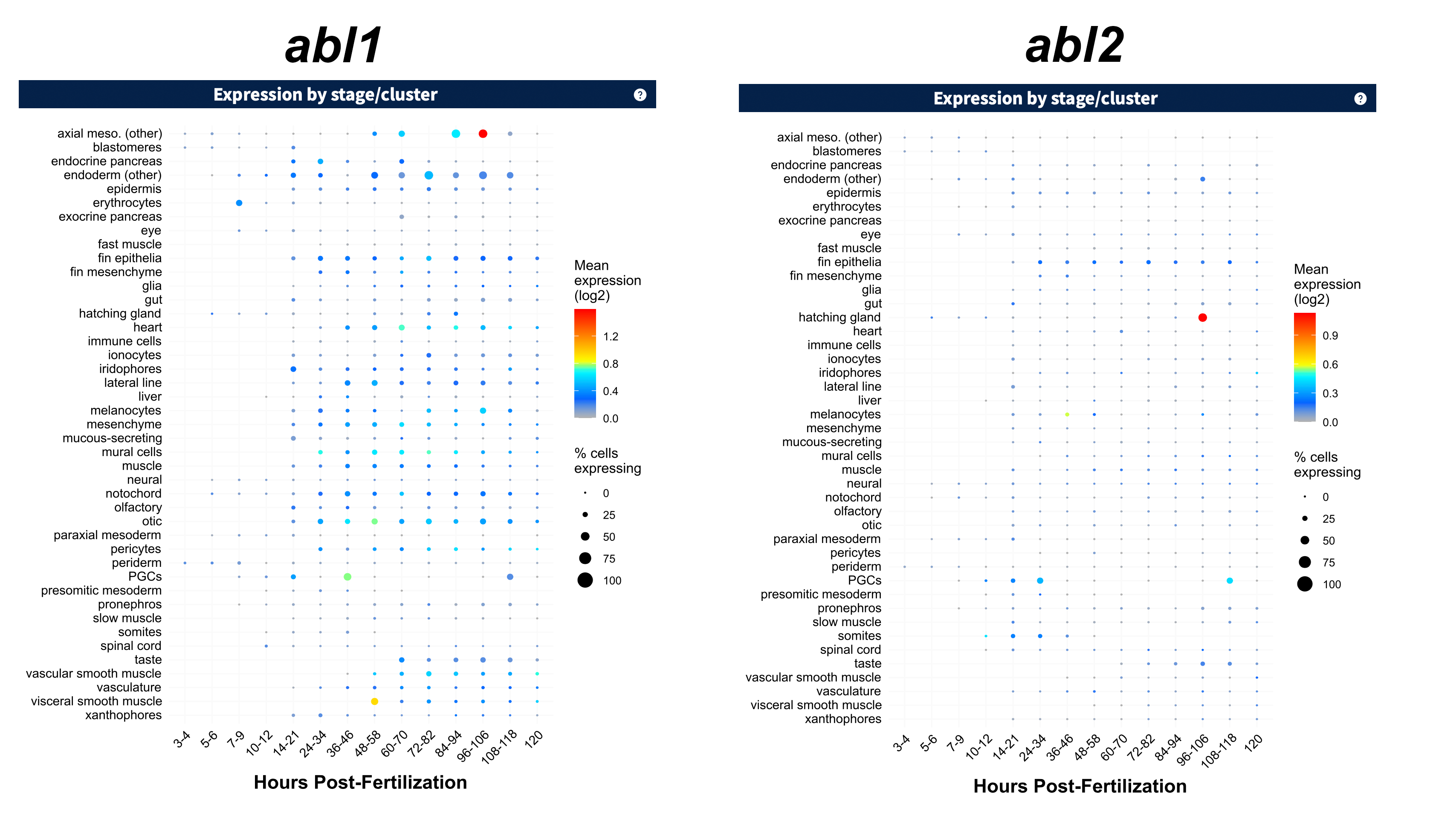

Supplement: S8 Fig — Data were generated by Daniocell atlas https://daniocell.nichd.nih.gov/ [31]. (TIF) [file pgen.1010851.s008.tif]

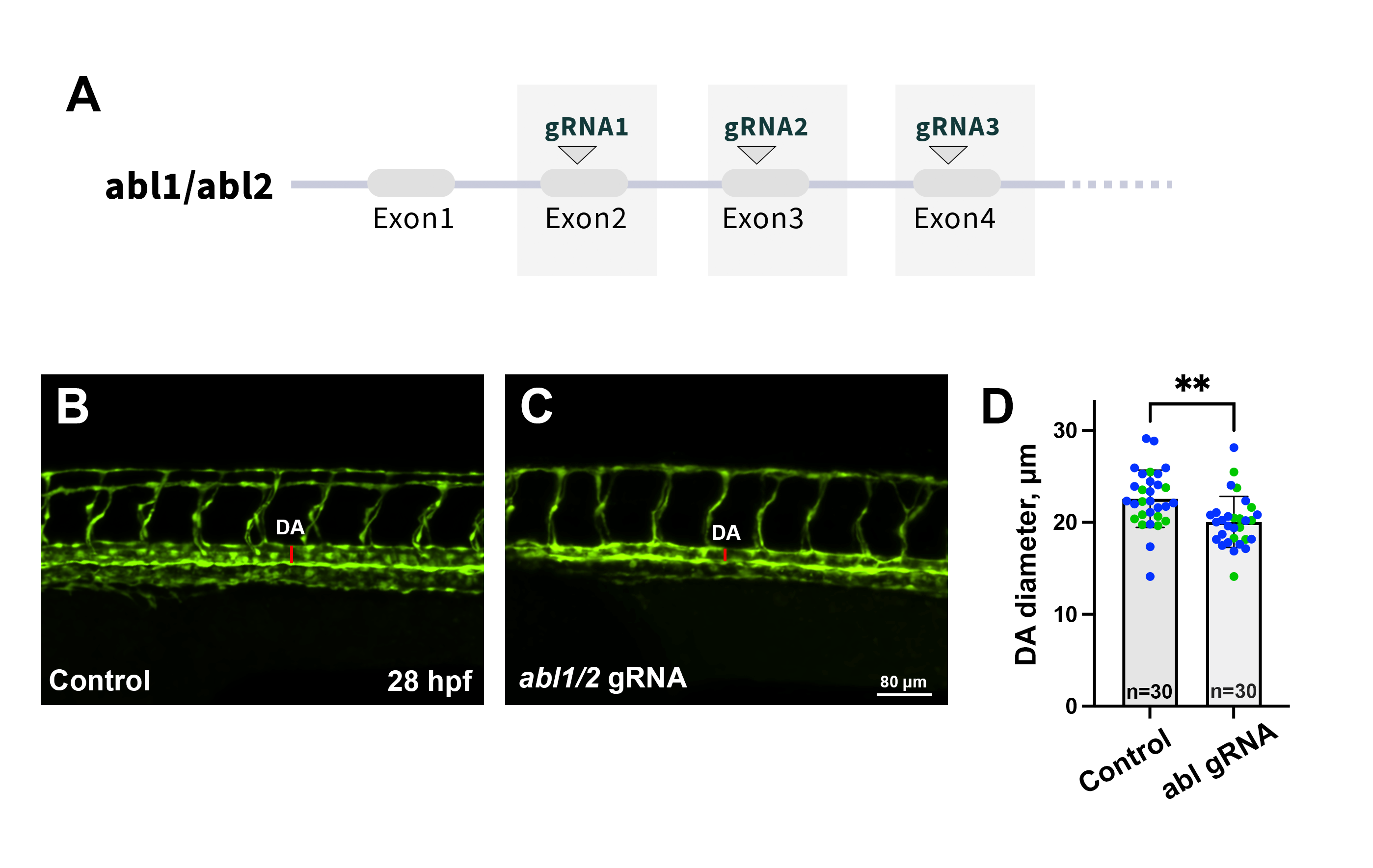

Supplement: S9 Fig — (A) A diagram illustrating the targeting sites of sgRNAs against abl1 and abl2 genes. Each gRNA was more than 90% effective based on DNA sequencing analysis. (B-D) Analysis of DA size in kdrl:GFP embryos at 28 hpf. Note the reduced DA diameter in embryos injected with abl1 and 2 gRNA mixture. Data from two replicate experiments are shown in different colors. Mean±SD is shown. **p<0.01, Student’s t-test. (TIF) [file pgen.1010851.s009.tif]

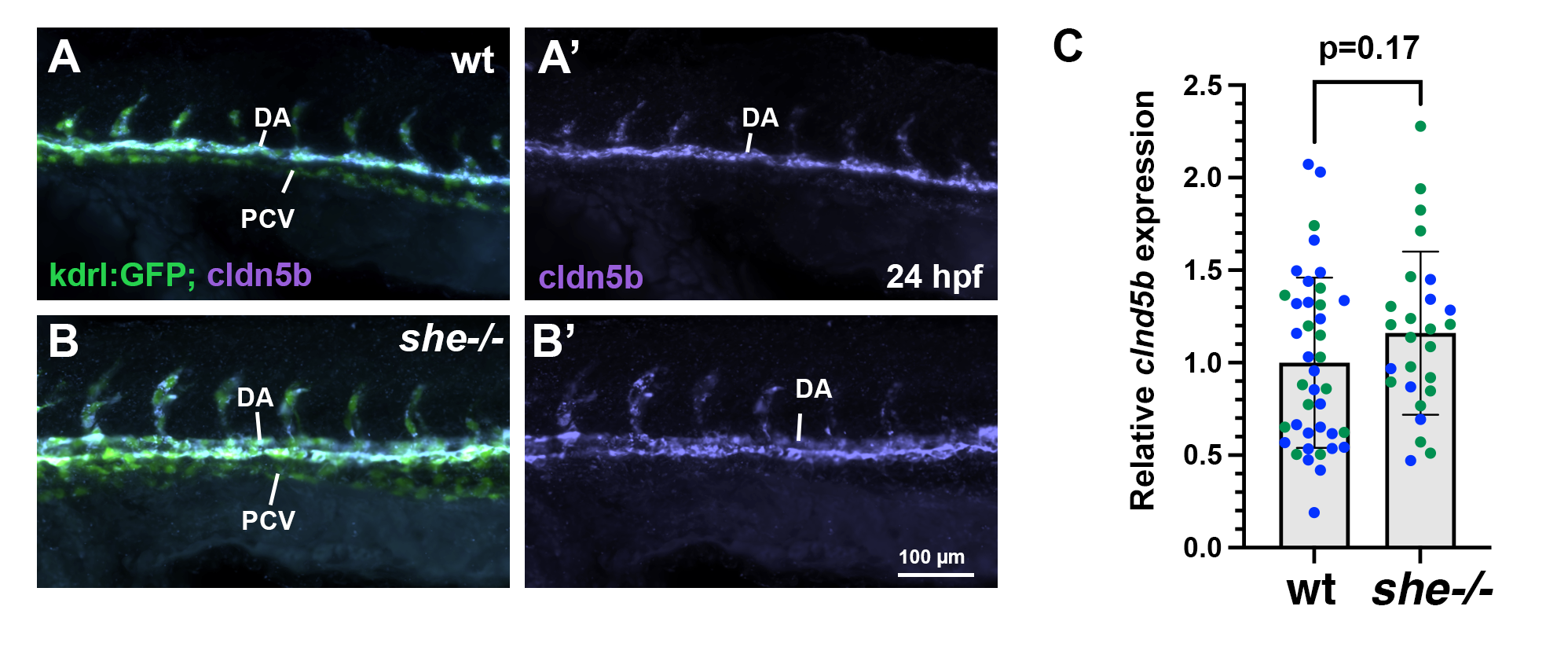

Supplement: S10 Fig — (A,B) cldn5b (purple) and kdrl:GFP fluorescence in the trunk region of she mutant and wild-type sibling embryos. DA, dorsal aorta; PCV, posterior cardinal vein. cldn5b fluorescence is shown in A’,B’. (C) Quantification of cldn5b fluorescence in the DA. p = 0.17, Student’s t-test. Error bars show SEM. Data show combined results from two independent experiments. (TIF) [file pgen.1010851.s010.tif]

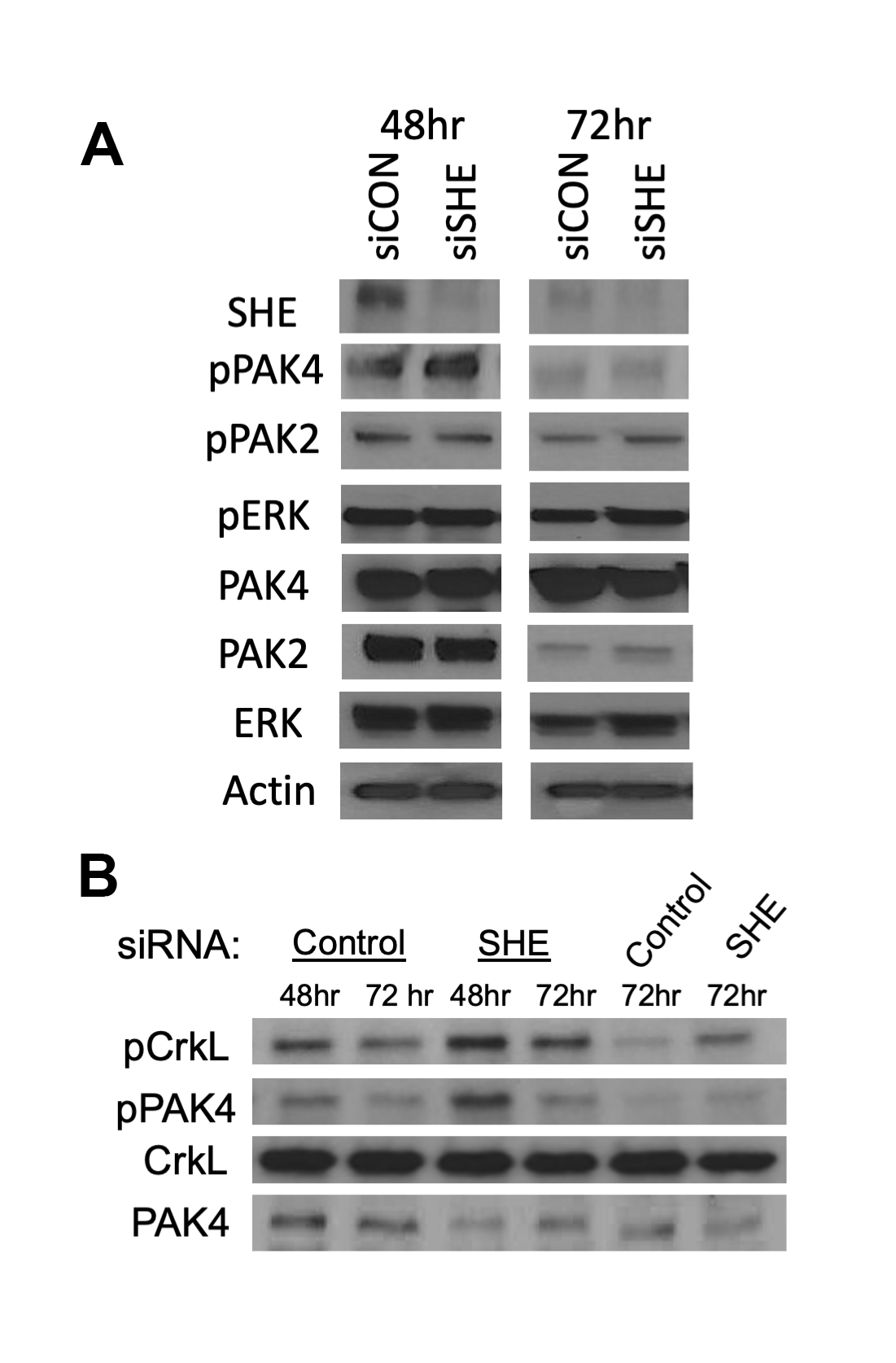

Supplement: S11 Fig — Replicate experiments for pPAK4 and pCRKL phosphorylation are shown in (B). Selected bands, which showed the greatest change, are displayed in the main Fig 8. (TIF) [file pgen.1010851.s011.tif]
